# Supramolecular Modification of Graphene Sponge with a Porphyrin Derivative Enhances the Photothermal Conversion Efficiency of a Solar Steam Generator

**DOI:** 10.1021/acsami.4c11299

**Published:** 2024-10-29

**Authors:** Elif Erçarıkcı, Demet Demirci Gültekin, Ezgi Topçu, Züleyha Kudaş, Murat Alanyalıoğlu, Kader Dağcı Kıranşan

**Affiliations:** a Department of Chemistry, Science Faculty, Atatürk University, Erzurum 25240, Turkey; b Department of Chemical Technology, Vocational School of Technical Science, Atatürk University, Erzurum 25240, Turkey; c Vocational School, Department of Food Processing, Bilecik Şeyh Edebali University, Bilecik 11100, Turkey

**Keywords:** solar energy, clean water, graphene sponge, porphyrin, gradient structure

## Abstract

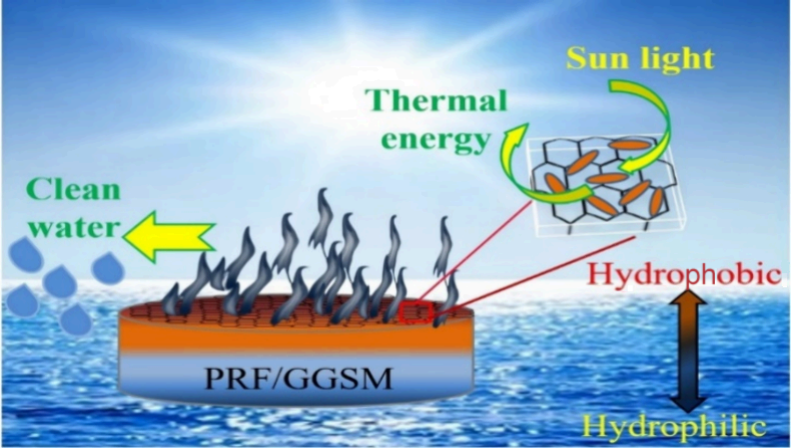

Solar energy seems to be a promising solution for obtaining clean water from saltwater and wastewater. With the solar steam generator system, it is possible to effectively acquire clean water from wastewater with a low-cost, sustainable, and environmentally friendly approach. In this study, PRF/GGSM, prepared by modification of gradient graphene sponge material (GGSM) with porphyrin derivative supramolecules (PRF), was investigated as a photothermal material for solar steam generation. PRF/GGSM possessing graphene and PRF served as ideal solar thermal converters that could easily gather sunlight. This material owing to its microporous and gradient hydrophilic structure has achieved a solar thermal conversion efficiency of up to 92% under 1 sun, corresponding to the water evaporation rate of 3.8 kg h^–1^ m^–2^. Moreover, this study exhibited that PRF/GGSM can efficiently generate clean water from seawater, wastewater, and even concentrated acid and alkali solutions.

## Introduction

With the rapid developments and population growth observed in modern societies, the increase in water pollution and the gradual decrease in freshwater resources have become a serious problem.^1,2^ Although many methods have been developed to overcome this problem, solar steam generators appear as a renewable and sustainable strategy to obtain clean water from seawater and water containing heavy metals and dyes.^3,4^ Since solar energy is an abundant and clean resource, it has been accepted as one of the most competitive energy sources that will solve water scarcity in the future. The conversion of solar energy into heat energy has a very high efficiency compared to other systems such as photovoltaics and photocatalysis.^5,6^ Recently, there has been intense interest in the production of water vapor from solar energy for its potential applications such as sterilization, water purification, and hygiene systems.^7^ The primary focus for the conversion of solar to thermal energy is to design new photothermal materials that are biodegradable, sustainable, and low cost and have high solar steam production efficiency.^8,9^

To obtain high thermal efficiency from materials designed for solar steam generators (SSG), they should first be light enough to float in water. Morphologically, materials consisting of either a porous structure^10^ or regularly arranged layers^11^ have low density. Second, these materials should have a hydrophilic character, which allows evaporated water to be transported through the material. Hydrophilic materials include wood,^12^ bacterial cellulose,^13^ natural latex,^14^ cellulose nanofibers,^15^ corn straw,^16^ poly(ethylene glycol) diacrylate,^17^ polyacrylamide aerogel,^18^ and polyurethane foam.^19^ Although these materials exhibit very effective performance in transporting water due to structurally hydrophilic properties, they lack high photothermal efficiency, which is the third important point for SSG. For this reason, the surfaces of these hydrophilic materials have been modified with various structures such as graphene, graphene oxide, carbon nanotube,^10,12,13,19−22^ polydopamine,^23^ polyaniline,^16,17^ MoS_2_,^24^ polypyrrole,^14,25^ and Au–CuS,^26^ which provide high light absorption and photothermal conversion. Such two-layer (Janus) materials exhibit a high rate of photothermal conversion; however, their preparation requires quite complex processes, so the effort to develop new materials for SSG continues unabated.

Apart from Janus structure materials, graphene is among the most used materials for SSG due to its high light absorption and photothermal activity. Three-dimensional graphene sponge materials (GSM) have attracted great attention in the scientific community for SSG due to their large specific surface area, lightness, flexibility, high mechanical strength, good thermal conductivity, and high photothermal efficiency.^27^ It is reported that GSM converts sunlight into heat energy at a high rate, but due to its hydrophobic character, it is insufficient to transport water to the upper parts, and therefore, the thermal efficiency is low (compared to Janus materials).^7,28^ In our previous study,^29^ GSM was prepared in a gradient structure in which the hydrophobic property gradually decreased from one end to the other, and this gradient material exhibited higher thermal efficiency and evaporation performance compared to GSM.

Supramolecules, which have come to the fore with their structural and functional properties in recent years, are a collection of chemicals consisting of different molecules that have been in the field of chemistry for many years.^30^ Compared to traditional organic substances, supramolecules provide light absorption at a wide wavelength due to their flexible and numerous π-conjugated systems. The photophysical or photochemical process of a supramolecule is initiated by the absorption of light, leading to an electronic excited state. The excited state is unstable because it has excess energy and prefers one of the molecular processes known as radiative emission (fluorescence or phosphorescence relaxation), vibrational relaxation (heat), or intersystem transfer. Among these processes, any nonradiative process leads to the conversion of light energy into heat energy.^30^ Since all these processes are competitive, photothermal conversion efficiency increases, especially if fluorescence, intersystem transfer, and photochemical reactions are inhibited.^31^ Reducing the fluorescence activities enables the photothermal properties of supramolecules to be increased; for this purpose, for instance, the supramolecule can be adsorbed on a solid support material. Thus, it has been reported that this property of supramolecules, which exhibit high fluorescence activity in a solution and free state, decreases by 80% when adsorbed to the surfaces of solid materials such as graphene oxide (GO)^32^ and boron nitride^33^ (via π–π or electrostatic interactions).^34^ Since there is no electron transfer after adsorption, the supramolecule mostly reflects the absorbed energy as photothermal energy. Porphyrin (PRF) derivatives (PRF-dS) are among the supramolecules whose photothermal properties have been investigated. PRF-dS form numerous absorption bands in both the near-IR and UV–visible regions.^35^ Photothermal transformations of such molecules are examined in various applications such as catalysis, micromotors, and actuators, especially in biomedical applications.^36^ Yan et al. synthesized the supramolecule (peptide–porphyrin nanorod (PPP-NDs)) with PRF polypeptides and applied PPP-NDs in antitumor therapy due to its high photothermal conversion feature and achieved successful results in photothermal therapy.^37^ PRF-dS exhibit high rates of photothermal conversion, making it important to investigate the use of these materials in SSG.

In our previous study, a flexible, durable, hydrophilic gradient-structured graphene sponge material (GGSM) was prepared.^29^ Here, PRF/GGSM was obtained by the adsorption of PRF to the hydrophobic region of GGSM to develop a more effective SSG system. The steam generation performance of PRF/GGSM, as a photothermal material in SSG, is increased about 3 times compared to GGSM due to both the graphene structure and PRF’s ability to absorb sunlight at wide wavelengths and high photothermal conversion. As a result, PRF/GGSM efficiently produced clean water with a removal efficiency of up to 97% from salt water and wastewater.

## Experimental Section

### Preparation of GGSM

GSM was prepared from GO suspension^38^ by the freeze-drying method as reported in our previous paper.^29^ Briefly, 20 mL of GO aqueous dispersion containing ascorbic acid (AA, 400 mg) as a reductant was vigorously stirred after the addition of sodium dodecyl sulfate (SDS) (50% by weight) for foaming. The resulting foamy dispersion was treated at 75 °C for 1 h, and the graphene hydrogel sponge (GHS) was obtained. GHS was frozen at −18 °C for about 5 h and then cooled naturally to room temperature. Subsequently, the as-prepared GHS was dried at 90 °C and then washed several times with ethanol and deionized water to remove excess AA. Finally, free-standing GSM was fabricated by annealing GHS at 350 °C.

GSM at the height of 1.2 cm cylindrical shape was impregnated with an acidic solution of H_2_SO_4_ /HNO_3_ (1:1) from the bottom part; thus, the oxidation of GSM has gradually carried out the acid treatment for an optimum 5 min.^29^ This material was washed several times with distilled water, and then GGSM was obtained after drying under atmospheric conditions. In GGSM, which has a height of about 12 mm, the part treated directly with acid between 0 and 4 mm is called the first region (R1), between 4 and 8 mm the second region (R2), and finally between 8 and 12 mm the third region (R3).

### Synthesis of PRF

PRF was synthesized,^31^ and the experimental procedure, nuclear magnetic resonance (NMR) (Figure S1), MALDI-TOF mass (Figure S2) spectrum, and chemical structure (Figure S3) of the resulting product are analyzed and presented in the Supporting Information. In summary, 5,10,15,20-tetrakis(4′-bromophenyl) porphyrin (0.16 g, 0.16 mmol) dissolved in 100 mL of chloroform was added to Zn(OAc)_2_·2H_2_O (53 mg, 0.24 mmol) dissolved in methanol by stirring in a N_2_ atmosphere for 24 h at room temperature. After the reaction, a dark-violet zinc-tetrabodipiporphyrin product was obtained. Boronate ester-BODIPY (80 mg, 0.16 mmol), zinc-tetrabodipiporphyrin (20 mg, 0.02 mmol), K_2_CO_3_ (0.22 g, 1.6 mmol), and Pd(dppf)_2_Cl_2_ (5 mg, 0.007 mmol) were mixed, toluene (6 mL) and water (1.0 mL) were added to the mixture and stirred for 18 h at 110 °C, and a dark violet-colored ZnPB_4_ solid was obtained. The ZnPB_4_ molecule was treated with HCl in dioxane for metal removal. After acid treatment, a NaHCO_3_ solution was used to neutralize. Then, the obtained solid was dried with CaCl_2_ to obtain the F_6_B_4_P supramolecule.^31^

### Preparation of PRF/GGSM

Although GSM is hydrophobic, the R1 and R3 sides of GGSM have high hydrophilic and hydrophobic characters, respectively.^29^ The materials for SSGs are expected to have high water transport capacity, solar absorption, and good photothermal conversion performance. Meanwhile, in Janus structure materials for SSGs, hydrophilicity in the material facilitates water absorption and transportation, while the hydrophobic character contributes to the photothermal efficiency.^29^ Modification of GGSM with PRF solution was performed from the R3 part of the material so that the hydrophobic PRF solution is both better immobilized and exhibits effective photothermal activity by interacting with direct sunlight. Since the R1 side exhibits a hydrophilic character, it enables water to be transported to the upper parts of the material more effectively and contributes to increasing vapor generation performance.

For the modification of GGSM, a 0.5 mg/mL PRF solution in dichloromethane (DCM) was prepared, and the GGSM (1.2 cm by height) was immersed 2 mm high from the hydrophobic parts in a supramolecule solution for different periods. Thus, 5 mg of PRF was adsorbed to GGSM. The concentration of the supramolecule solution and the retention time in the solution were optimized. The experimental procedure for the preparation of PRF/GGSM and the drying of PRFs after adsorption to GGSM is presented in Figure 2 and Video S1, respectively.

### Characterizations

The morphology and structure of PRF/GGSM were investigated by field emission scanning electron microscopy (FESEM, ZEISS SIGMA 300) equipped with energy-dispersive X-ray spectroscopy (EDX). Fourier transform infrared (FTIR) spectroscopy was performed for the structural analysis of the samples by using a PerkinElmer spectrometer. Optical characterizations of PRFs were analyzed using a UV–vis–NIR spectrophotometer (Shimadzu 3101PC). The crystal structure of the samples was analyzed by powder X-ray diffraction (XRD) using a Rigaku Mini Flex X-ray diffractometer with Cu Kα radiation (λ = 1.5406 Å). The microstructure of PRF dispersion was investigated by transmission electron microscopy (TEM, Hitachi HT7700). Agilent Cary Eclipse Brand Fluorescence was used to examine the fluorescence properties of PRFs. The atomic ratios of elements in PRF/GGSM were characterized using X-ray photoelectron spectroscopy (XPS, Spect-Flex spectrometer), equipped with a monochromatic Al Kα X-ray source. Raman spectra of the samples were attained from a Raman microscope (WITech alpha 300R) at an excitation laser wavelength of 532 nm.

### Steam Generation Experiments

Figure 5 shows the experimental system used for solar steam generation. The cylindrical PRF/GGSM samples (1.2 cm in height and 2 cm in diameter) were floated on 30 mL of water in a double-walled glass. The surface temperature and weight loss over the entire process were recorded by using an IR camera and an electronic mass balance, respectively. The simulated solar irradiation, power varying from 1 sunlight (1 kW m^–2^) to 10 sunlights (10 kW m^–2^), was provided using a OptaSense (OPT-S500F) Photocatalytic Xenon Light Source.

### Antibacterial Test

*Escherichia coli* cells were grown in Luria Broth (LB) medium for 12 h at 37 °C. Before the antibacterial test, *E. coli* cells were harvested in the exponential growth phase by centrifugation at 6000 rpm for 3 min. PRF/GGSM was first soaked in 75% alcohol for 30 min and then washed with phosphate buffer solution. After 5 mL of an *E. coli* suspension (10^6^ CFU/mL) was added to PRF/GGSM in a sterilized Petri dish, the whole sample was placed in an incubator shaker. The colony counting assay method was applied at regular intervals for 24 h to determine the amount of changes in *E. coli* bacteria.

## Results and Discussion

### Photochemical and Morphological Characteristics of PRF

**Figure 1:**
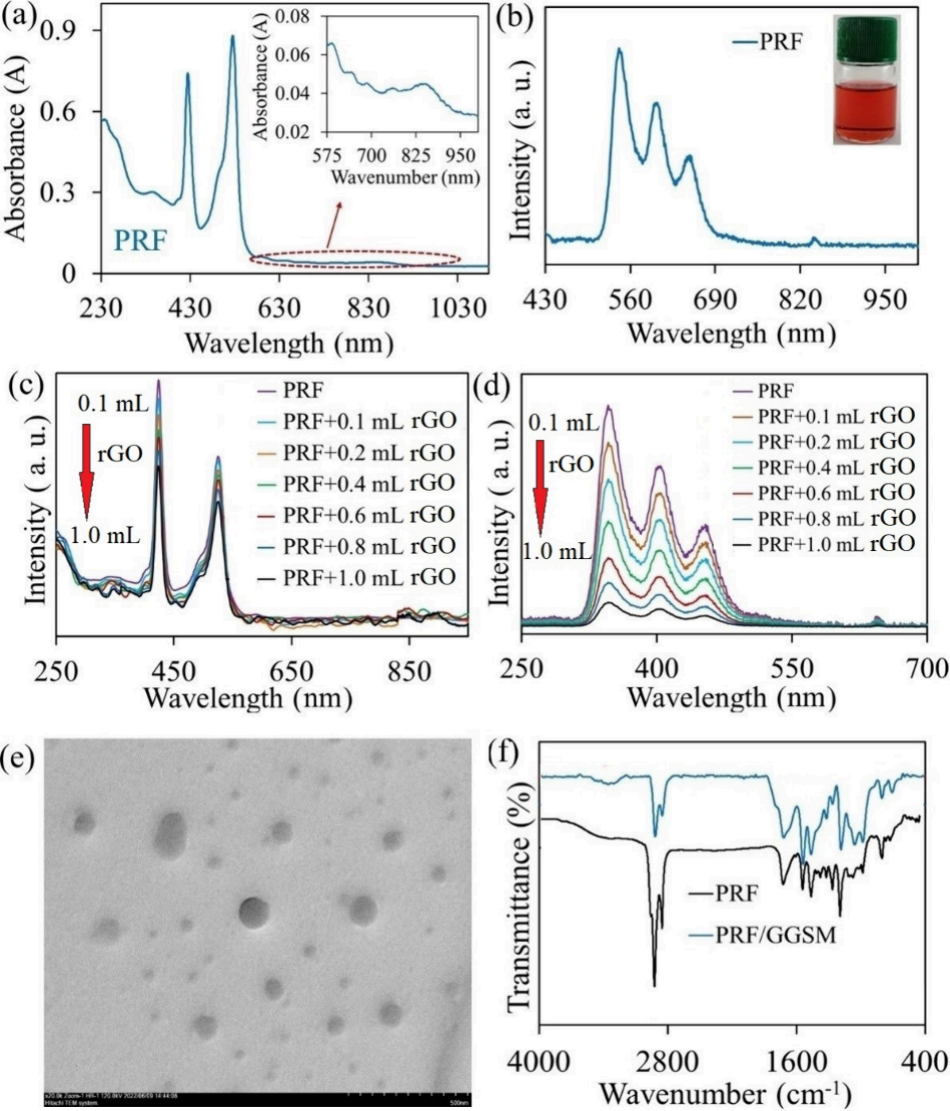
UV–visible absorption spectrum (a) and fluorescence spectrum (b) of PRF. UV–visible absorption (c) and fluorescence spectrum (d) of solutions containing different volumes of rGO dispersion and PRF. TEM image of PRF (e). FTIR spectra of PRF and PRF/GGSM (f).

The absorption properties of the synthesized PRF were determined by using UV–visible absorption spectroscopy (Figure 1a).

The UV–vis spectrum of PRF exhibited several absorption bands at a wavelength range of 900 to 230 nm, with maximum absorption peaks at 430 and 530 nm. The intense peaks at 527 and 425 nm and the shoulder at 352 nm are Soret bands corresponding to the S_0_ → S_2_ transition in π–π* electronic excitation. The weak peaks in the range of 500–750 nm are Q bands corresponding to the S_0_ → S_1_ transition in π–π* electronic excitation.^39^ The fluorescence peaks of PRF at 652, 602, and 544 nm in Figure 1b are the mirror image of the Soret peaks in the UV–vis absorption spectrum. The wavelengths of the peaks at 527, 425, and 352 nm in Figure 1a are red-shifted to 17, 177, and 300 nm, respectively, due to the Stokes shift in the fluorescence spectrum (Figure 1b).^39^

The interactions of PRF with sponge materials composed of reduced graphene oxide (rGO) were investigated by using UV–visible absorption and fluorescence spectroscopy. Increasing volumes of rGO dispersion were added to the PRF solution, and due to the adsorption of supramolecules to the surface of graphene layers through π–π interactions, both the absorption and fluorescence intensities of the prepared mixtures decreased as shown in Figure 1c,d.^32^ After 1.0 mL of rGO dispersion was added to PRF, there were decreases of about 25% in absorption intensity and 92% in fluorescence intensity, indicating that PRF can relax with a high rate of thermal conversion, as it cannot release the absorbed energy as fluorescence. Therefore, this absorbed energy is mostly released as heat energy, which is concluded by generating highly efficient steam.

The morphological structure of PRF was investigated using TEM in Figure 1e. TEM images of PRF exhibited sphere-like crystal structures with sizes ranging from 50 to 100 nm.

### Optimization of Preparation Parameters of PRF/GGSM

**Figure 2:**
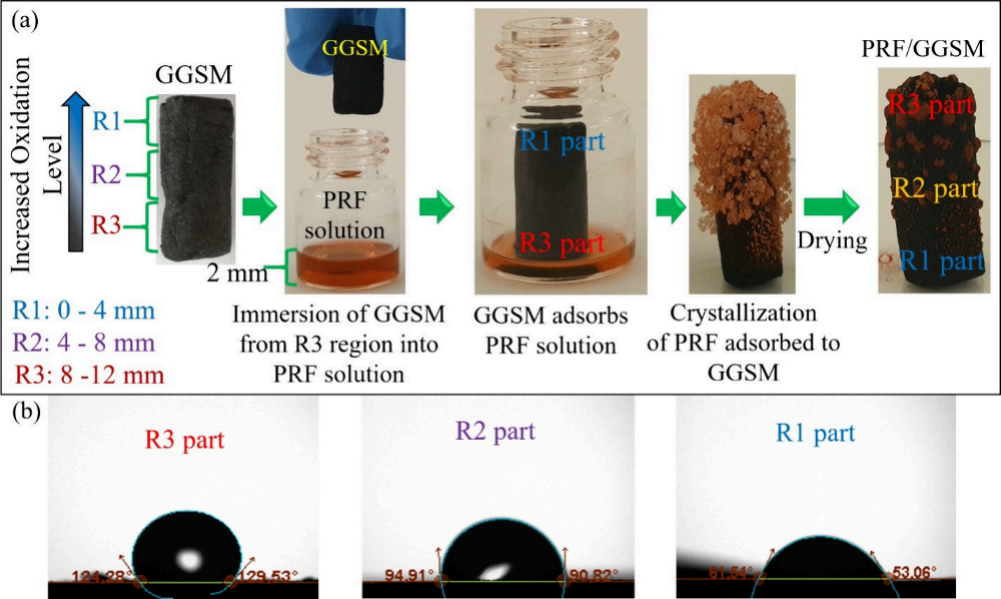
(a) Preparation procedure of PRF/GGSM. (b) Water contact angle measurements of the R3, R2, and R1 of PRF/GGSM.

According to the experimental procedure in Figure 2, the immobilization of PRF onto the GGSM surface was examined by using FTIR spectroscopy. FTIR spectra of PRF/GGSM and PRF are presented in Figure 1f. The spectrum of GGSM exhibited a broad peak for O–H vibration (at 3000–3600 cm^–1^), aromatic C–H vibration peak (at 2967 cm^–1^), and C=O, COOH, and C–O–C (at 1733, 1624, and 1386 cm^–1^, respectively).^29,40^ The FTIR spectrum of PRF showed an N–H stretching vibration at 3325 cm^–1^, C–H stretching in the pyrrole ring at 2954 cm^–1^, and aliphatic C–H vibrations at 2921 and 2851 cm^–1^. In addition, for the C=N bending vibration in the porphyrin ring at 1701 cm^–1^, peaks corresponding to C–N stretching, in-plane N–H, and out-of-plane N–H vibrations were recorded at 1376, 979, and 752 cm^–1^, respectively.^41^ The peaks for both PRF and GGSM in the FTIR spectrum of PRF/GGSM indicate that GGSM has been successfully modified with PRF. Thus, PRF can adsorb to the GGSM surface through π–π interactions due to the π-conjugated systems in the structures of both GGSM and supramolecules.^32^

For the gradient modification of the hydrophobic surface of GGSM with PRF, first, the concentration of the supramolecule solution (Figure S4a) and immobilization time (Figure S4b–d) were optimized by FTIR analyses. When the concentration of the PRF solution is lower than 0.5 mg/mL and the immobilization time is less than 2 min, C–H (2291 cm^–1^), C=N (1701 cm^–1^), and N–H (1376 cm^–1^) vibrations of PRF were not observed (Figure S4). Therefore, the optimum concentration for PRF to be adsorbed as a gradient starting from the hydrophobic part of GGSM was determined to be 0.5 mg/mL and the immobilization time was 2 min. In the digital camera image of PRF/GGSM prepared under the optimum conditions in Figure 2a, it is clear that PRF is gradually adsorbed on the GGSM surface.

Water contact angle measurements of the GGSM are presented in Figure S5. The water contact angles of the R3, R2, and R1 of GGSM decreased from about 124.1 to 61.5° in parallel with the increase in hydrophilic character.

After modification of GGSM with PRF, it was determined with water contact angle measurements of three regions (R3, R2, and R1) that GGSM retained its gradient (in terms of hydrophilicity–hydrophobicity) feature (Figure 2b). A water contact angle of 124.3° was measured in R3, which has a hydrophobic character, and that of 62.5° in hydrophilic R1. There was no significant difference in water contact angles after the modification of GGSM with PRF. While the contact angle increased in R3 and R2 that interacted directly with PRF, which has a partially hydrophobic character, no significant difference was observed in R1.

From these results, it was predicted that PRF/GGSM would be very effective in transporting water to the upper parts, especially with its gradient structure.

### Morphological and Structural Characterization of PRF/GGSM and GGSM

**Figure 3:**
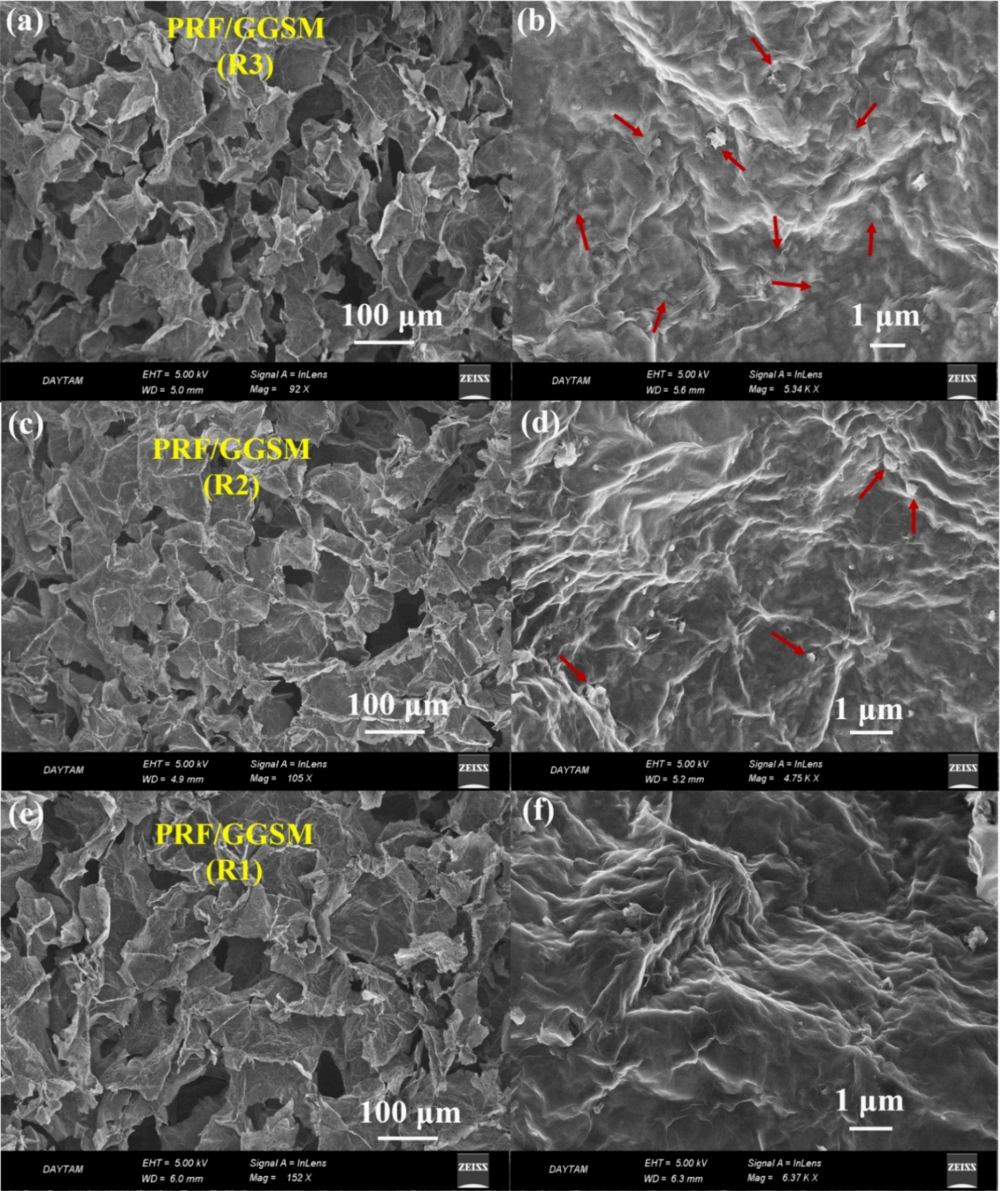
FESEM images at different magnifications for (a, b) R3, (c, d) R2, and (e, f) R1 of PRF/GGSM.

Figure 3 depicts FESEM images of different regions of PRF/GGSM prepared under the optimum conditions. In Figure 3a,b, in addition to the porous structure of GGSM, rounded PRFs are observed in R3. Since PRF is gradually modified onto GGSM, PRF is partially observed in R2 (Figure 3c,d) and not at all in R1 (Figure 3e,f).

**Figure 4:**
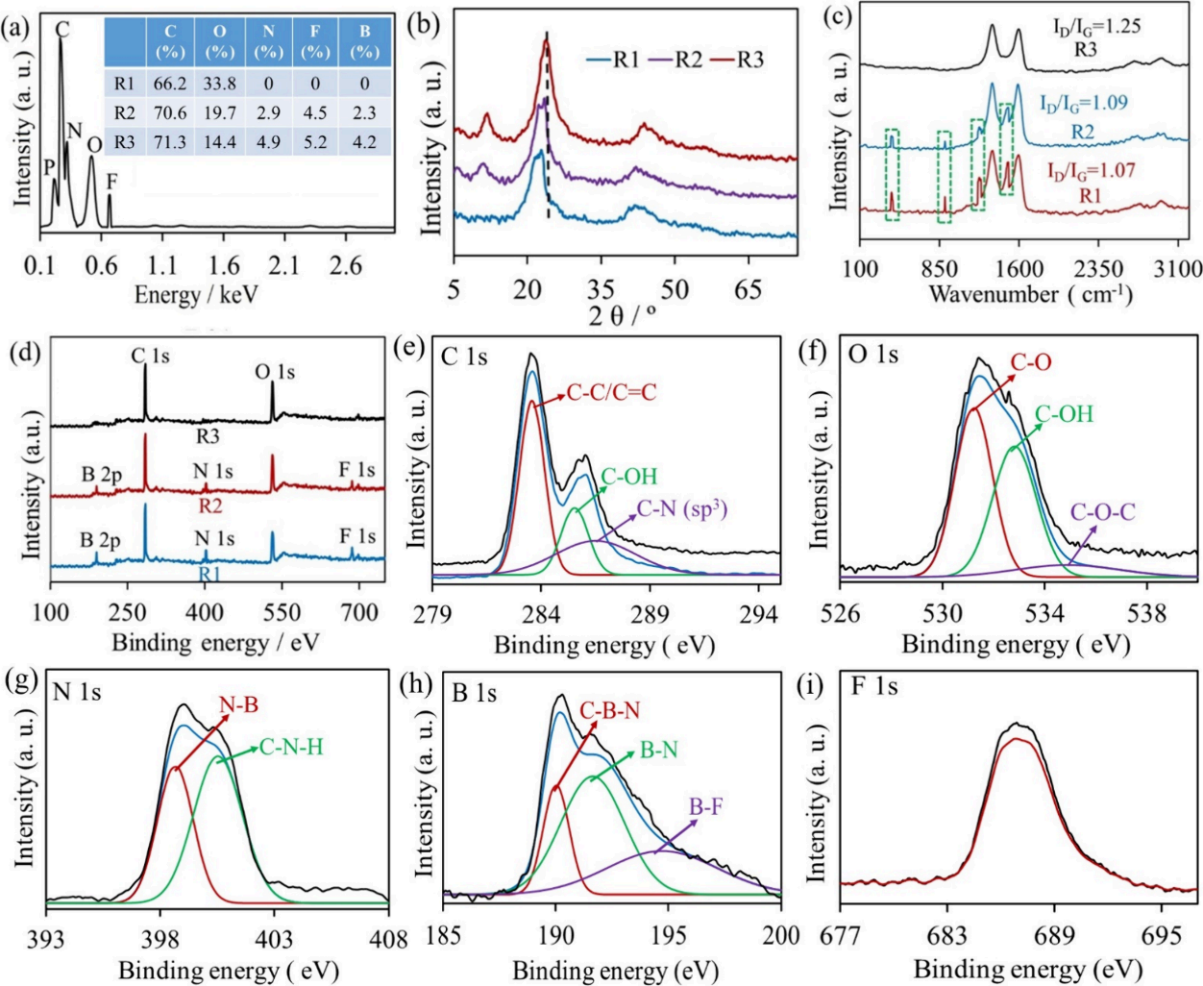
(a) EDX spectrum for the R3 of PRF/GGSM. Inset: table of the % atomic ratios of the R3, R2, and R1 of PRF/GGSMs. XRD (b), Raman (c), and XPS (d) spectra for the R1, R2, and R3 of PRF/GGSM. Deconvoluted XPS spectra of (e) C 1s, (f) O 1s, (g) N 1s, (h) B 1s, and (i) F 1s of PRF/GGSM.

EDX of PRF/GGSM shows that the atomic ratios of N, F, and B for R3 are higher than those of R2, and there are only C and O atoms for R1, confirming the gradient structure of PRF/GGSM (Figure 4a).

Figure 4b demonstrates the XRD spectra of three different regions of PRF/GGSM. The R3 of PRF/GGSM shows only a characteristic diffraction (002) peak of graphene at 24.6°. However, for R2 and R1, in addition to the (002) diffraction as in the GGSM structure,^29^ there is a peak of GO at 10.6° due to oxidation. For R3 and R2, where PRF is densely adsorbed between interlayers, the (002) diffraction peak shifted to smaller 2θ values, attributing the increment of the distance between the graphene layers in the GGSM to PRF.^42^

Raman spectra show the G (at 1597 cm^–1^, originated by the plane vibration of the graphene ring) and D (at 1360 cm^–1^, due to defects in the graphene layers) bands, which are the characteristic of the graphene sponge (Figure 4c). Additionally, for R3 and R2, the peaks at 409 and 903 cm^–1^ correspond to out-of-plane and in-plane bending deformation of the phenyl rings in the PRF structure, respectively. The Raman spectrum of R2 and R3 also exhibits two extra peaks assigned to the symmetric stretching of C–N–C and C–N(H)–C bonds and the relatively weak asymmetric stretching of the C–C–C bonds of the phenyl rings (at 1240 cm^–1^) and the asymmetric stretching of the C–C–NH bonds and relatively weak symmetric C–C–C bond stretching (at 1478 cm^–1^).^43^ It is supposed that the supramolecules have been adsorbed to the defected regions densely in PRF/GGSM. This case can be confirmed with a decrement in *I*_D_/*I*_G_ value, indicating the patch role of adsorbed highly conjugated molecules onto the graphene structure (Figure 4c).^44^

Figure 4d presents the XPS spectrum of the R1, R2, and R3 of PRF/GGSM. From R3 to R1, the intensity of the C peak decreased and that of the O peak increased (Figure 4d). Further, the peaks of N, B, and F atoms, not seen in R1, were recorded in R2 and R3, confirming that PRF is gradually adsorbed to GGSM. Considering the atomic ratios for the 3 regions (Table S1), while there are only C and O atoms originating for graphene in R1, from R2 to R3, the ratio of N, F, and B atoms gradually increased because of the intensity of the PRF adsorption. The deconvolution of the C 1s peak for the R3 of PRF/GGSM is presented in Figure 4e. C 1s displays three peaks at 283.5, 285.5, and 286.7 eV for the C–C/C=C, C–OH, and C–N bonds, respectively.^45^ The O 1s of R3 includes three peaks for C–O (at 531.3 eV), C–OH (at 533.1 eV), and C–O–C (at 535.4 eV) in Figure 4f.^46^ For N 1s (Figure 4g), two typical peaks of N–B and N–C–H bonds are located at 398.8 and 400.5 eV, respectively.^47^ The B 1s region (Figure 4h) exhibits three peaks at 190, 191.8, and 195 eV for C–B–N, B–N, and B–F bonds, respectively.^48^Figure 4i shows the position of the F 1s peak centered around 686.9 eV.^49^ These results confirmed that PRF was successfully adsorbed to the GGSM structure.

### Amount of Water Absorption of PRF/GGSM

Fast and effective absorption of water through the material is one of the main parameters affecting the high-performance operation of SSGs. Cylindrical PRF/GGSMs (0.6 cm in diameter and 2 cm in height) were immersed in a 4.0 mM rhodamine B (Rh B) aqueous solution from the R3 part. The time-dependent movement of the dyed water was monitored with a camera, and it is presented in Video S2. PRF/GGSM initially absorbed water very quickly, but due to the gradient PRF in the structure, water absorption decreased with time. The water absorption time for PRF/GGSM is 40 s. The weight (η_w_, %) and volume (η_V_, %) swelling ratios of PRF/GGSM measured at different temperatures were determined using eqs S1 and S2, respectively (Table S2).^17^ As a result of the experiments, the water absorption rate of PRF/GGSM was calculated as 50.68 kg m^–3^ h^–1^, which is higher than the steam production rate under 1 sunlight. Rapid water absorption is of great importance for the continuity of the process as it prevents drying during steam production.

### Thermal Conductivities of PRF/GGSM

The thermal conductivity of PRF/GGSM was determined with eq S3, as explained in detail in the Supporting Information. Since thermal insulation plays an important role in trapping heat on the evaporation surface during photothermal conversion, the thermal conductivity of PRF/GGSM in both wet and dry states was investigated and calculated using thermal camera images of PRF/GGSM (Figure S6 and Table S3).^12,14^ This photothermal material has a much lower thermal conductivity value than that of water (0.61 W m^–1^ K^–1^) due to the porous structure of the graphene sponge serving as an effective thermal barrier to retain heat on the evaporating surface during solar steam generation.

### Photothermal Performance of PRF/GGSM

**Figure 5:**
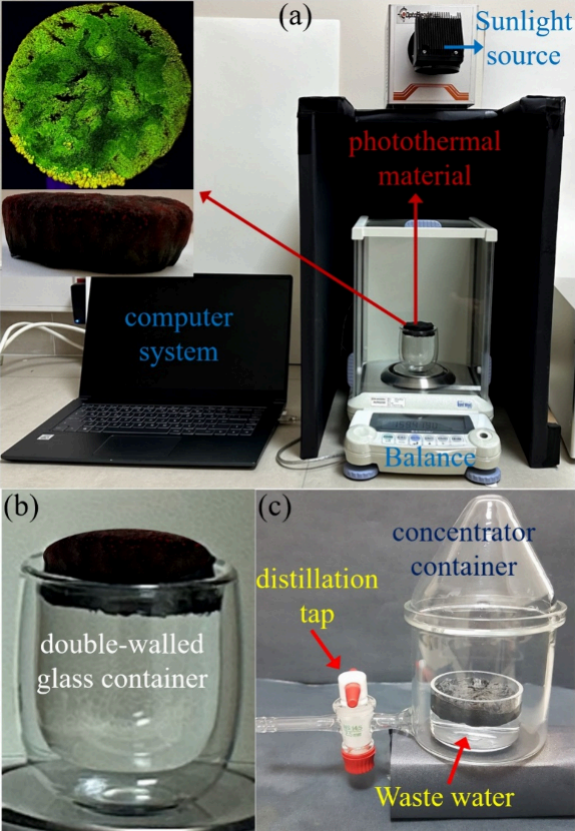
(a) Water evaporation by PRF/GGSM (inset: photographs of PRF/GGSM under UV light and daylight). (b) Double-walled glass container including wastewater and PRF/GGSM. (c) Device of solar distillation for clean water production.

**Figure 6:**
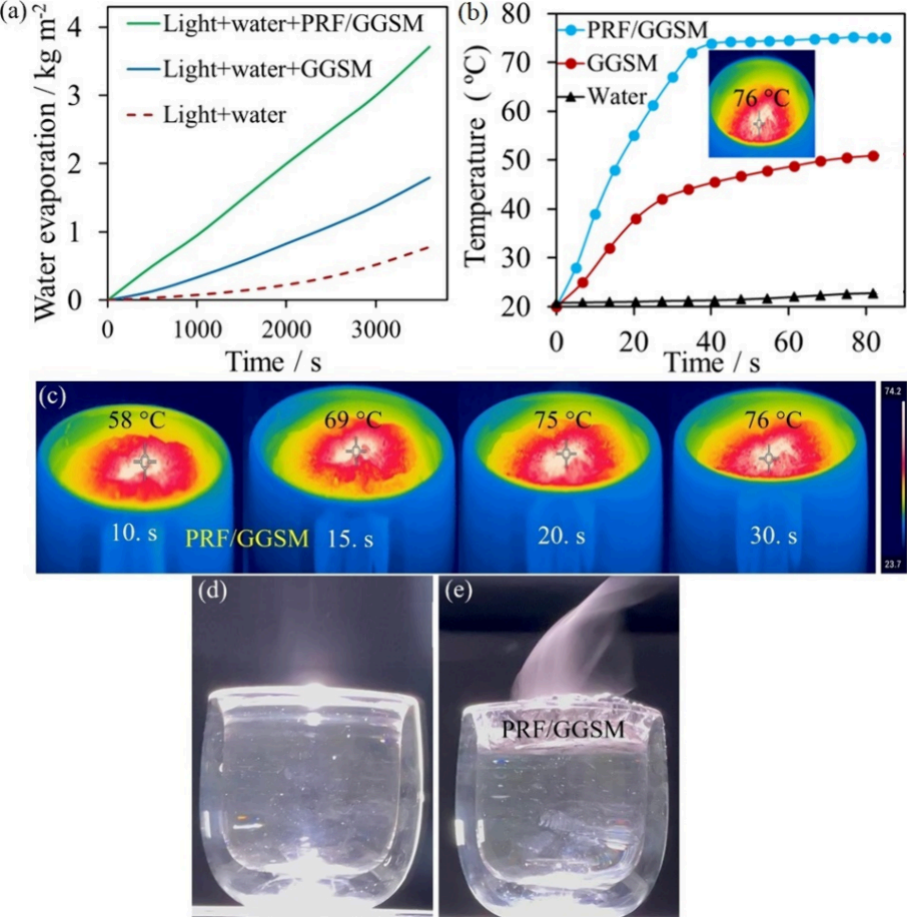
(a) Amount of water evaporation with (green line) and without (red dashed line) PRF/GGSM. (b) Temperature during 100 s of irradiation of water and PRF/GGSM. (c) Thermal camera images after different irradiation times for PRF/GGSM. The photographs of (d) water and (e) PRF/GGSM at the 40th second of irradiation under sunlight.

The use of PRF/GGSM as a photothermal material in solar steam generator applications was investigated with the experimental setup in Figure 5a. The image of PRF/GGSM under UV light demonstrates that the top surface of the material is homogeneously modified with PRF. In addition, the gradient modification of PRF onto PRF/GGSM can be seen in the inset of Figure 5a. The water evaporation rate for PRF/GGSM was determined using the system in Figure 5b. During the experiments, the R1 of the gradient material was placed toward water. The evaporation performance after 1 h for PRF/GGSM was determined as 3.7 kg/m^2^ h (Figure 6a). The evaporation rate of the material modified with the supramolecule increased about 2 times compared to GGSM (1.8 kg/m^2^ h, Figure 6a),^29^ attributed to the high rate of light absorption of the supramolecule and the conversion of absorbed light to thermal energy. In addition, the high photothermal efficiency of PRF is based on the fact that electrons are easily delocalized due to the regular conjugated system, and the B–N-coordinated covalent bond relaxes by reducing the rigidity and converting the energy.^50,51^ Furthermore, the contribution of F atoms in the structure of PRF to electron transfer increases the photothermal efficiency.^37^

Figure 6b shows the change in surface temperature of water and PRF/GGSM during 100 s of irradiation. The surface temperatures (20 °C) increased for water to 23 °C, for GGSM to 50 °C, and for PRF/GGSM to 76 °C after 80 s of irradiation, which can be attributed to the photothermal activity of both graphene and the PRF structure (Figure 6b,c). Video S3 demonstrates the surface temperature depending on the irradiation time of PRF/GGSM. The period for the PRF/GGSM surface to reach the maximum temperature is 40 s. This high increase for PRF/GGSM (from 20 to 76 °C) in such a short time is due to the high photothermal efficiency of PRF.

Figures 6d,e shows the photographs of water with and without PRF/GGSM under sunlight. After the 40th second of irradiation, unlike in Figure 6d, dense microscopic water droplets suspended in the air due to rapid evaporation on the water surface form a cloud-like appearance in Figure 6f. This image on the water surface was attributed to rapid steam generation due to the photothermal property of PRF (Video S4).

The evaporation enthalpy for PRF/GGSM was calculated by eq S5, and solar vapor conversion efficiency was determined (by eq S4) as 96%. Such a high evaporation efficiency is attributed to the sunlight absorption ability of the supramolecule and its conversion to thermal energy. In addition, it can be considered that PRF increases vapor efficiency by acting as a barrier, preventing heat loss on the GGSM surface.

### Heat Losses for the Designed SSG System

**Figure 7:**
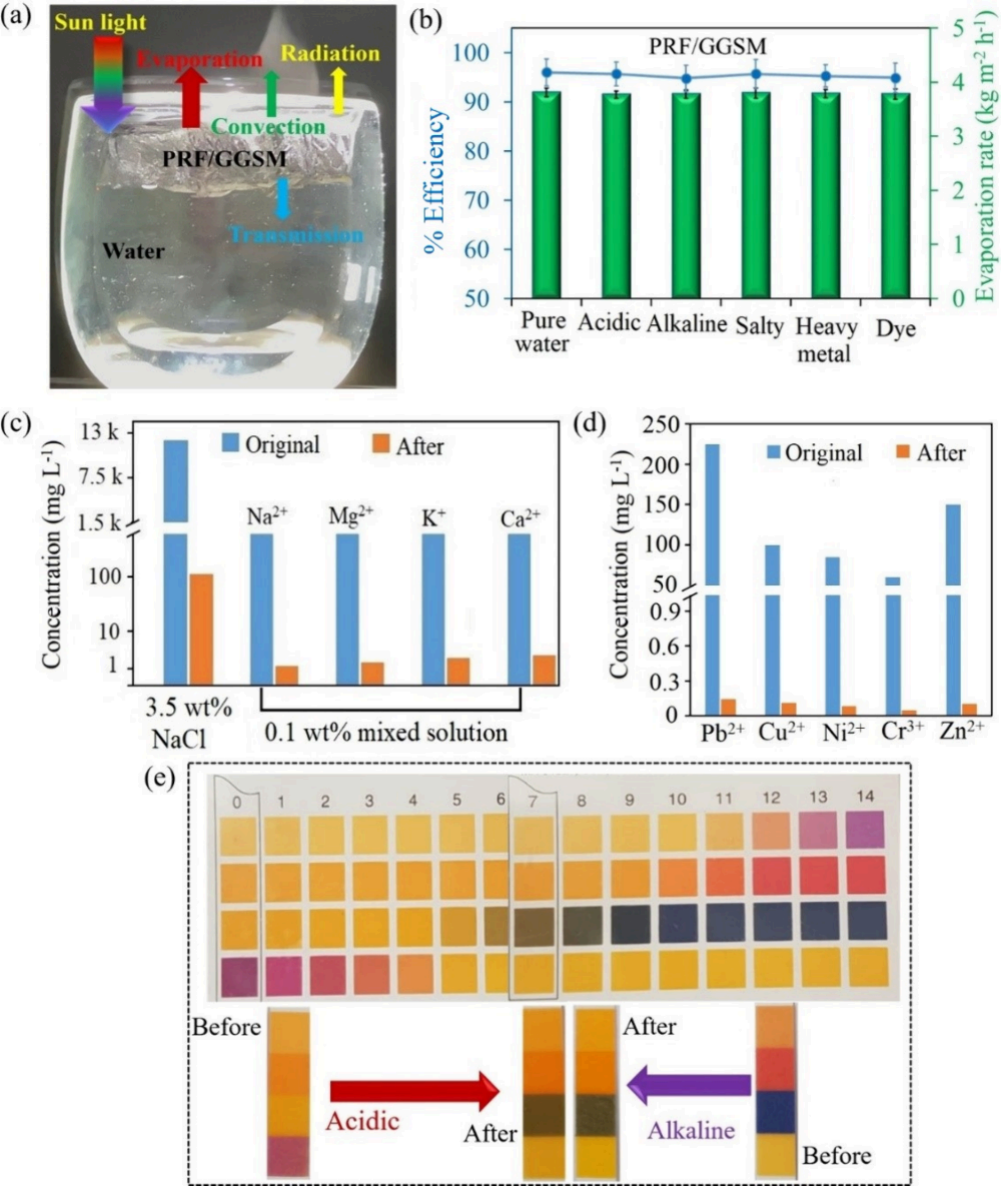
(a) Energy balance and heat transfer diagram for PRF/GGSM in steam generation. (b) Water evaporation rate and solar thermal conversion efficiency of PRF/GGSM in different solutions after 1 h under 1 sunlight. After distillation with PRF/GGSM, the changes in ion amounts in (c) 3.5% NaCl solution and solution containing 0.1% Na^+^, K^+^, Mg^2+^, and Ca^2+^ ions (*k*: 1000), (d) 225 mg/mL Pb^2+^, 100 mg/mL Cu^2+^, 90 mg/mL Ni^2+^, 75 ppm of Cr^3+^, and 150 mg/mL Zn^2+^. (e) Images of pH values before and after solar thermal purification of the acidic (pH 2) and alkaline (pH 12) solutions with PRF/GGSM.

In the SSG system, heat is lost in four different ways. These are evaporation, radiation, convection, and conduction, schematically displayed in Figure 7a.^28^ The equations to calculate the heat loss are shown in the Supporting Information, and the results are presented in Table S4. PRF/GGSM converted absorbed sunlight into steam at a high rate. Radiation, convection, and conduction losses are low in PRF/GGSM, attributed to the fact that the supramolecule wraps the GGSM surface like a protective cover, minimizing thermal losses and converting solar energy into thermal energy with high efficiency.^52^

### Solar Thermal Conversion Efficiency of PRF/GGSM at Different Water Contents

The evaporation rate and solar thermal conversion efficiency of PRF/GGSM in different water contents (pure water, acidic (pH 2), and basic (pH 12) solutions and solutions containing separately 3.5% NaCl, 100 mg/mL Cu^2+^, and 1.0 g/L methyl orange) were investigated, and the results are depicted in Figure 7b and Table S5. For PRF/GGSM, the % standard deviations of the evaporation rate and solar thermal conversion efficiency were calculated as 3.43 and 0.42, respectively. These close values revealed that PRF/GGSM could work well in saltwater and wastewater, besides concentrated acid and alkali solutions, promising for applications in the photothermal regeneration of clean water.

### Desalination with PRF/GGSM

Two separate solutions containing 3.5% NaCl and 0.1% Na^+^, K^+^, Mg^2+^, and Ca^2+^ ions were prepared, and desalination experiments were performed with PRF/GGSM. The ion contents before and after distillation with PRF/GGSM were determined by ICP-MS, and the results are shown in Figure 7c and Table S6. Initially, 1275 ppm of Na^+^ was detected in saltwater and 100 ppm of Na^+^ ions were detected after purification with PRF/GGSM. Table S6 demonstrates the ion content of the solution containing 0.1% Na^+^, K^+^, Mg^2+^, and Ca^2+^ ions before and after distillation. Desalination performance for PRF/GGSM was determined as 92%. The high desalination activity of this photothermal material can be attributed to the easy electrostatic attraction between positively charged ions in saltwater and the negatively charged regions (functional groups such as hydroxyl, epoxy, and carbonyl) in the porous sponge structure.

The times for obtaining 10 mL of distilled water were determined as 4.0 and 8.2 h for PRF/GGSM and GGSM, respectively (Table S7). Such a low time for PRF/GGSM can be attributed to supramolecules generating more vapor per unit of time and having higher vapor generation efficiency, as expected.

Desalination experiments were carried out with Black Sea and Mediterranean marine water, and the results are presented in Table S8. Considering the Na^+^ ion in seawater, PRF/GGSM achieved desalination of 99.8 and 99.9% in Mediterranean and Black Sea seawater, respectively. These results revealed that PRF/GGSM produces clean water from seawater as a promising SSG material.

### Purification of Wastewater Containing Heavy Metals with PRF/GGSM

Heavy metal ions in drinking water cause chronic poisoning by inactivating proteins and enzymes in the human body. For this reason, the removal of heavy metals from drinking water is of great importance. Solar thermal purification experiments of solutions containing 225 mg/mL Pb^2+^, 100 mg/mL Cu^2+^, 90 mg/mL Ni^2+^, 75 ppm of Cr^3+^, and 150 mg/mL Zn^2+^ were carried out with PRF/GGSM (Figure 7d). The heavy metal removal performance and the time for the purification of a 10 mL solution were determined as 98% (Table S9) and 4.2 h (Table S7) for PRF/GGSM, respectively. The time to distill the same volume of water with GGSM is 8.4 h, which is attributed to the fact that the PRF-containing material generates more steam per unit time, thus achieving a higher steam generation efficiency, as expected. These results showed that the purification of wastewater containing heavy metal ions is successfully achieved with PRF/GGSM.

### Purification of Acidic and Alkaline Solutions with PRF/GGSM

To test PRF/GGSM in the purification of concentrated acidic/basic solutions, solar thermal purification was carried out with pH 2 and pH 12 solutions. The acidic and alkaline solutions reached neutral pH after solar thermal purification with PRF/GGSM (Figure 7e). It is suggested that removal in the acidic solution was achieved by the electrostatic attraction between H_3_O^+^ ions and the negatively charged functional groups of the graphene-based material. The purification in alkaline solution is achieved through the adsorption of OH^–^ ions to the pores in the sponge structure. The results revealed that PRF/GGSM is an effective photothermal material for the purification of the acidic/basic solutions.

### Purification of Wastewater Containing Dye with PRF/GGSM

**Figure 8:**
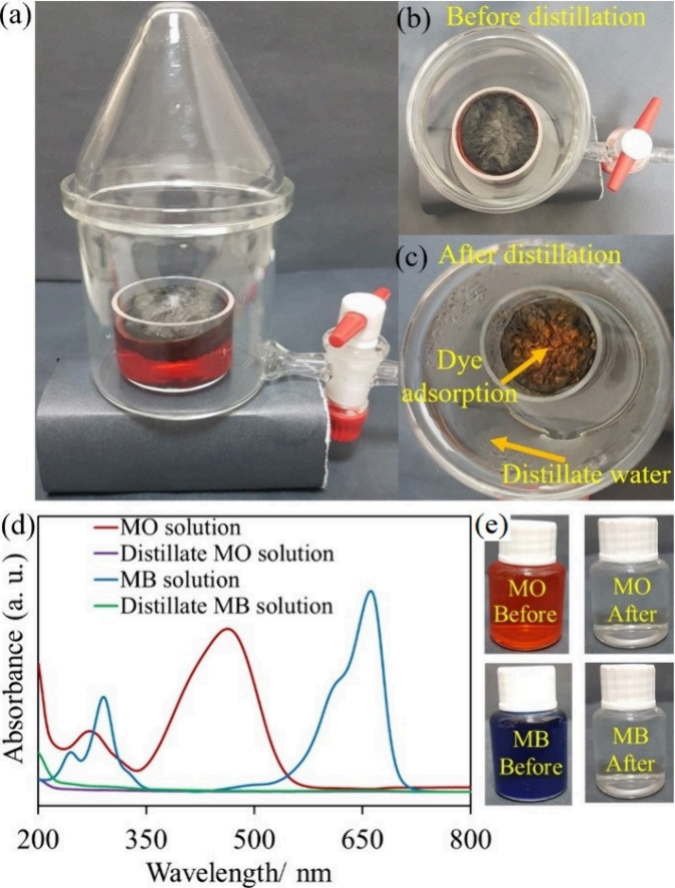
(a) Solar distillation for clean water production from dye solution with PRF/GGSM. Photographs of the distillation system before (b) and after (c) solar purification of the MO solution. (d) UV–visible absorption spectra and (e) photographs of the MO and MB dye solutions before and after solar purification.

The solar purification performance of PRF/GGSM was investigated in dye solutions containing 1.0 g/L methyl orange (MO) and methylene blue (MB). These dyes have a positive charge and a regular π-conjugated system (Figure S7a,b). The distillate of the solutions after under 10 sun irradiation for 2 h was examined by UV–visible absorption spectroscopy (Figure 8d). After solar purification with PRF/GGSM, the UV–visible absorption spectrum of the distillates was very similar to that of pure water. In addition, photographs of both dye solutions before and after distillation displayed clear purification of dyes, which was acquired by adsorption to sponge surfaces through both π–π interactions and electrostatic interactions between dyes and graphene.^53^ Further, photographs of the PRF/GGSM surface before and after distillation (Figure 8b,c) show that the MO dye accumulated on the material surface. Distillation of the MO solution with PRF/GGSM is shown in Video S5. The time to obtain 10 mL of clean water from dye solutions with PRF/GGSM was determined as 3.9 h. PRF/GGSM achieved distillation in a shorter time compared to GGSM (7.9 h) due to its high steam generation rate (3.4 kg/m^2^ h).

### Antibacterial Property of PRF/GGSM

The antibacterial property of PRF/GGSM was tested by using Gram-negative species of *E. coli*. The results of bacterial counts performed for 24 h are presented in Figure S8. Since no material existed in the control group, bacteria continued to multiply. On the other hand, in the presence of PRF/GGSM, the amount of bacteria decreased by 25%, which is attributed to the partially antimicrobial effect of PRF.

### Photothermal Properties and Stability of PRF/GGSM under Daylight

**Figure 9:**
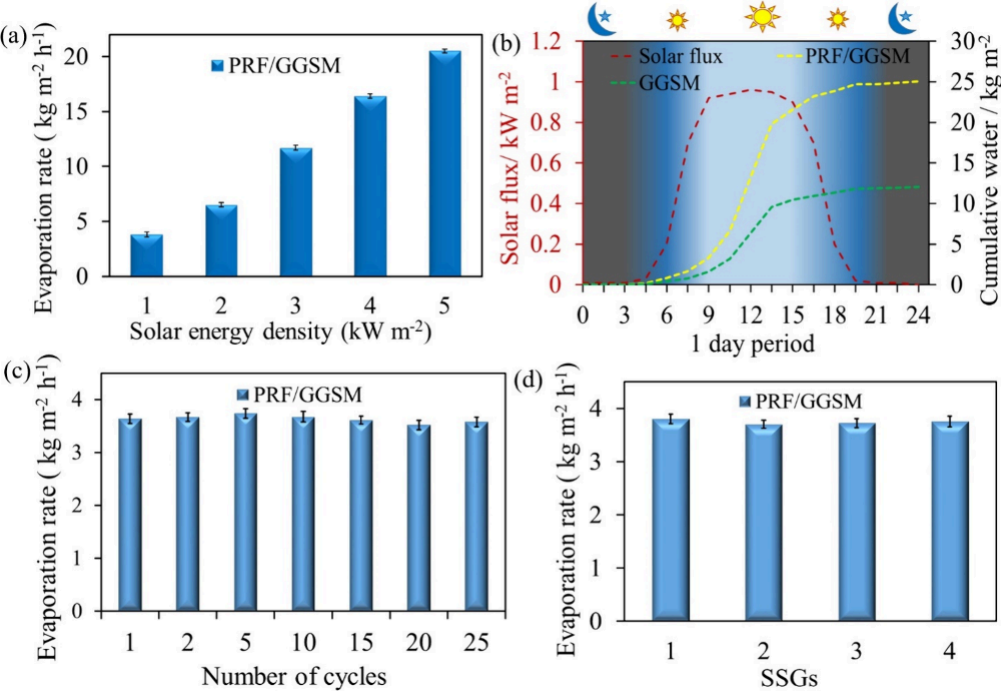
(a) Evaporation rate of PRF/GGSM and corresponding solar energy density. (b) Amount of distilled water with PRF/GGSM depending on the solar flux during the day. (c) Evaporation rate of PRF/GGSM after 25 cycles of utility. (d) Evaporation rate of PRF/GGSM prepared at different times using the same method.

Figure 9a shows the evaporation rate for PRF/GGSM depending on the solar energy intensity. The evaporation rates of this material increase proportionally as the solar energy intensity increases, indicating that it is sensitive to the solar energy intensity. PRF/GGSM was used as a photothermal material in the open-air system, shown in Figure S9. The amount of water collected against the solar flux during 1 day is recorded in Figure 9b. Experiments were carried out in Erzurum-Turkey (average temperature: 25 °C; humidity: 58%; sunrise 04:50, sunset 19:20) in July. The solar flux and the amount of distilled water increased with sunrise and decreased near sunset. The results under daylight were close to the evaporation amounts obtained under 1.0 kW cm^–2^ sunlight under laboratory conditions, indicating that our photothermal material can also be used in SSG applications under daylight.

### Long-Term Stability and Reproducibility of PRF/GGSM

To examine the long-term stability of the materials, solar evaporation was repeated for 25 cycles using the same material. In each cycle, the water-wetted and swollen material was removed from the system and water was removed by mass pressure. The materials were dried at 60 °C for 20 min and reused in the next cycle. There was only a 2.7% decrease in the initial evaporation rate after the 25th cycle, thanks to the structure of graphene and the photothermal stability of PRF (Figure 9c). Four PRF/GGSMs were prepared at different times using the same method, and each of them was used as the photothermal material for SSG. PRF/GGSMs have very similar evaporation rates (Figure 9d), demonstrating that materials with the same structural and chemical properties can be obtained by using the proposed preparation procedure.

To test the stability of PRF absorbed on GGSM, the EDX spectrum and the atomic ratios obtained after the 1st, 5th, and 15th cycles of utilization are presented in Figure S10. For the EDX spectrum, adsorbed salt ions were not taken into account to determine whether PRF remained on the surface during the process. It was observed that there was no significant change in atomic ratios after the 1st, 5th, and 15thcycles, and thus, PRF remained stable on the GGSM surface.

The SSG performance of PRF/GGSM is compared with those of different materials (Table S10). Most of the materials in Table S10 have a Janus structure and thermal losses that may occur due to two different structures, reducing vapor generation performance and efficiency. On the other hand, PRF/GGSM exhibits high solar thermal conversion efficiency thanks to the high light absorption and photothermal efficiency of both graphene and PRF, as well as longer-term stability. Moreover, our material demonstrates effective performance in repeated uses due to the gradual immobilization of PRF to GGSM.

## Conclusions

In summary, we developed a graphene sponge with a porous structure modified with a supramolecule. The structural and morphological characterization of PRF/GGSM showed that PRF was gradually adsorbed to GGSM. Thanks to graphene and PRF, this material absorbs sunlight at a high rate and acts as an ideal solar thermal converter. PRF/GGSM exhibited a high water evaporation rate of 3.8 kg h^–1^ m^–2^ g^–1^ and a solar thermal conversion efficiency of 92%. Moreover, in the SSG system, PRF/GGSM achieved an effective purification performance (over 97%) under rigorous conditions, such as wastewater, strong acid, and alkali solutions. Compared with other materials for water regeneration, PRF/GGSM is much more efficient, lightweight, and portable and has a lower cost for the regeneration of clean water. Moreover, PRF/GGSM achieved a higher purification performance than GGSM, which is due to the modification of PRF possessing a high absorption ability and good photothermal activity. After all, the as-prepared material demonstrates great potential in various practical applications such as desalination, metal extractions, and wastewater treatment.

## Supporting Information Available

Adsorption, crystallization, and drying of PRF on the GGSM surface (MP4)
Water absorption with PRF/GGSM (MP4)
Surface temperature changes of PRF/GGSM depending on the irradiation time (MP4)
Cloud-like images resulting from dense water droplets suspended in the air due to vapor generated on the surface depending on the irradiation time of PRF/GGSM (MP4)
Generating clean water from dye solution with PRF/GGSM (MP4)
Figures and tables: porphyrin derivative supramolecular synthesis (F_6_B_4_P); NMR and MALDI-TOF mass spectrum of F_6_B_4_P; chemical structure of PRF; FTIR spectra of the R3, R2, and R3 of PRF/GGSM; water contact angle measurements for three different regions of GGSM; atomic % ratios of the R1, R2, and R3 of PRF/GGSM; weight (η_w_, %) and volume (η_V_, %) swelling ratios of PRF/GGSM; thermal conductivity of PRF/GGSM; solar thermal conversion efficiency of PRF/GGSM; heat losses for the designed SSG system; ion amount after distillation with PRF/GGSM; times obtained for 10 mL of distillate from solutions containing saltwater, heavy metal, and dye with PRF/GGSM and GGSM; change in the amounts of some ions in desalination with PRF/GGSM from seawater; heavy metal ion amounts detected after distillation with PRF/GGSM; chemical structure of MB and MO; antimicrobial activities of PRF/GGSM and GGSM; photograph of solar steam generator application under daylight; EDX spectrum after the 15th consecutive cycle of PRF/GGSM; comparison of PRF/GGSM with various materials on SSG performance under 1 sun (PDF)
