# Supplementary material for: Supramolecular Modification of Graphene Sponge with a Porphyrin Derivative Enhances the Photothermal Conversion Efficiency of a Solar Steam Generator
Source: ACS Appl Mater Interfaces. 2024 Oct 29;16(45):61910–20. doi: 10.1021/acsami.4c11299 (PMC11565480; doi:10.1021/acsami.4c11299)
Supplement: Supplementary file 6 — am4c11299_si_007.pdf [file am4c11299_si_007.pdf]

Supporting Information

Supramolecular Modification of graphene sponge with a porphyrin derivative enhances the photothermal conversion efficiency of a solar system generator

Elif Erçarıkcı[a], Demet Demirci Gültekin[b], Ezgi Topçu[a], Züleyha Kudaş[a], Murat Alanyalıoğlu[c]
and
Kader Dağcı Kıranşan*[a]

a Department of Chemistry, Science Faculty, Atatürk University, Erzurum, 25240, TURKEY
b Department of Chemical Technology, Vocational School of Technical Science, Atatürk University, Erzurum, 25240, TURKEY
c Bilecik Şeyh Edebali University, Vocational School, Department of Food Processing, Bilecik, 1100, TURKEY
kdagci@atauni.edu.tr

Porphyrin Derivative Supramolecular Synthesis (F_6B_4P):

Synthesis of 8-chlorophenyl-BODIPY: 3-Ethyl-2,4-dimethyl-1H-pyrrole (2.16 mL, 16 mmol) and 4-chlorobenzaldehyde (1.124 g, 8 mmol) were added to a 1 L round-bottomed flask and dissolved in dry CH_2Cl_2 (600 mL). TFA (10 drops) was added and the mixture was stirred at room temperature for 24 h. DDQ (1.82 g, 8 mmol) was added in a single portion, and the reaction was stirred at room temperature overnight. N,N-Diisopropylethylamine (16 mL, 91 mmol) and $BF_3 \cdot Et_2O$ (17 mL, 128 mmol) were added and the mixture was stirred at room temperature for 24 h. Then, washed with water (3 x 150 mL) and brine (3 x 150 mL). The combined organic layers were dried over Na_2SO_4 and concentrated in vacuo. The residue was purified by column chromatography (SiO_2, CH_2Cl_2) to afford 1 as a dark-violet solid (1.82 g, 55%).

1H NMR (400 MHz, CDCl3) δ ppm: 7.51 (d, J = 8.5 Hz, 2H), 7.26 (d, J = 8.5 Hz, 2H), 2.56 (s, 6H), 2.35 (q, J = 7.6 Hz, 4H), 1.34 (s, 6H), 1.00 (t, J= 7.5 Hz, 6H).

13C NMR (CDCl3, 100 MHz) δ ppm: 154.2, 138.5, 138.1, 134.9, 134.3,133.0, 130.6, 129.9, 129.4, 17.1, 14.6, 12.5, 11.9.

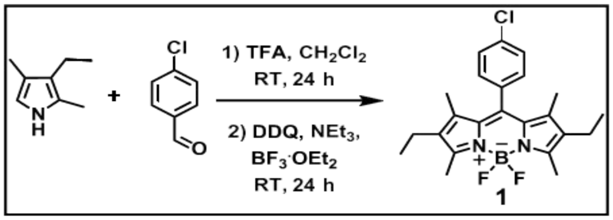

Reaction for the synthesis of 8-chlorophenyl-BODIPY.

Synthesis of boronate ester-BODIPY: Aliquots of 1 (0.10 g, 0.241 mmol), bis(pinacolato)diboron (0.19 mg, 0.75 mmol), $Pd_2(dba)_3$ (2.3 mg, 0.0025 mmol), XPhos (4.8 mg, 0.01 mmol), and KOAc (75 mg, 0.75 mmol) were added into an oven-dried 50 mL Schlenk tube. The Schlenk tube was capped with a rubber septum and then evacuated and backfilled with nitrogen three times. 1,4-Dioxane (4 mL) was added via syringe and the resulting mixture was deoxygenated by three freeze-thaw-pump cycles. The reaction mixture was heated to 110 °C under a nitrogen atmosphere for 24 h. Then, the reaction mixture was diluted with CH_2Cl_2 (50 mL) and washed with brine (3 x 100 mL). The organic layer was dried over Na_2SO_4, filtered and concentrated in vacuo. The residue was purified by column chromatography (SiO_2, petroleum ether/CH_2Cl_2, 50:50) to afford Boronate ester BODIPY as an orange solid (90 mg, 74%).

1H NMR (300 MHz, CDCl3) δ ppm: 7.82 (d, J = 7.8 Hz, 2H), 7.22 (d, J = 7.2 Hz, 2H), 2.45 (s, 6H), 2.24 (q, J = 7.5 Hz, 4H), 1.32 (s,12H), 1.19 (s, 6H), 0.91 (t, J = 7.5 Hz, 6H).

13C NMR ($CDCl_3$, 100 MHz) δ ppm: 152.6,139.0, 137.6, 137.3, 134.2, 131.6, 129.5, 126.5, 83.0, 28.6, 23.8, 16.0, 13.5, 11.4, 10.7.

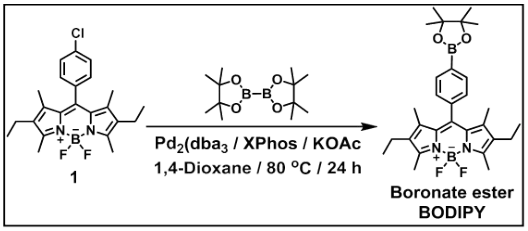

Reaction of boronate ester-BODIPY synthesis.

Synthesis of 5,10,15,20-tetrakis(4’-bromophenyl): A solution of 4-bromobenzaldehyde (3.7 g, 20 mmol) in propionic acid (150 mL) was refluxed at 140 °C. Pyrrole (1.38 mL, 20 mmol) was added dropwise and the reaction mixture was stirred at 140 ^{o}C for 2 h. Reaction mixture was cooled to room temperature and the resulting dark violet precipitate was collected by filtration and washed with hot water then with methanol (3 x 50 mL) The product was further purified by recrystallization from dichloromethane and methanol (1.95 g, 33%).

1H NMR (300 MHz, $CDCl_3$) δ ppm: 8.86 (s, 8H, β-pyrrolic-H), 8.09 (d, J = 8.3 Hz, 8H), 7.92 (d, J = 8.3 Hz, 8H).
13C NMR ($CDCl_3$, 75 MHz) δ ppm: 140.8, 135.8, 130.0, 122.6, 121.0, 119.0.

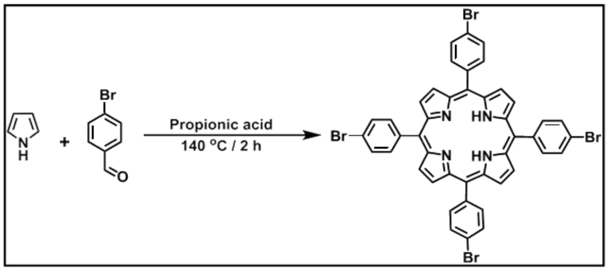

Reaction for the synthesis of 5,10,15,20-Tetrakis(4'-bromophenyl)porphyrin.

Synthesis of PB$_4$: Boronate ester-BODIPY (80 mg, 0.16 mmol) TetraBodipyporphyrin (20 mg, 0.02 mmol), K_2CO_3 (0,22 g, 1.6 mmol) and Pd(dppf)$_2$Cl$_2$ (5 mg, 0.007 mmol) were added into an oven-dried 10 mL Schlenk tube. The Schlenk tube was capped with a rubber septum and then evacuated and back-filled with nitrogen three times. Toluene (6 mL) and water (1 mL) were added via syringe and the resulting mixture was deoxygenated by three freeze-thaw-pump cycles. The reaction mixture was heated to 110 °C under a nitrogen atmosphere for 18 h. Then, the reaction mixture was diluted with CH_2Cl_2 (50 mL) and washed with brine (3 x 100 mL). The organic layer was dried over Na_2SO_4, filtered and concentrated in vacuum. The residue was purified by column chromatography (SiO_2, CH_2Cl_2 to CH_2Cl_2/MeOH 98:2) to afford PB4 as a dark-violet solid (6.5 mg, 15%).

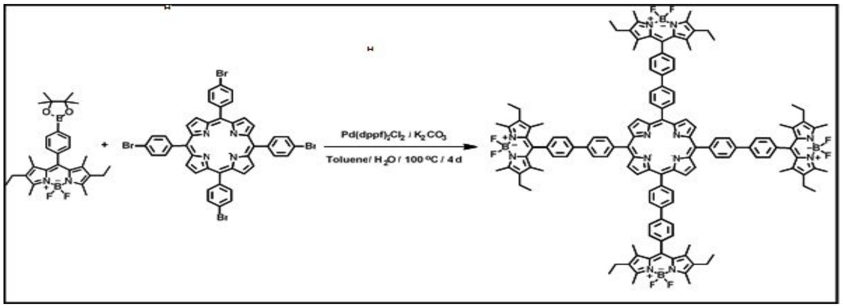

Reaction of F_6B_4P synthesis

^{1}H NMR (400 MHz, $CDCl_3$) δ ppm: 9.03 (s, 8H), 8.32 (d, J = 7.2 Hz, 8H), 8.10-8.02 (m, 16H), 7.46 (d, J = 7.6 Hz, 8H), 2.51 (s, 24H), 2.35-2.24 (m,16H), 1.19 (s, 24H), 1.02-0.90 (m, 24H).

^{13}C NMR ($CDCl_3$, 100 MHz) δ ppm: 154.1,150.4, 142.6, 141.2, 140.0, 139.3, 138.6, 135.4, 135.3, 133.1, 132.3, 131.0, 129.9,129.3, 127.9, 125.4, 121.0, 29.9, 17.3, 14.9, 12.2,

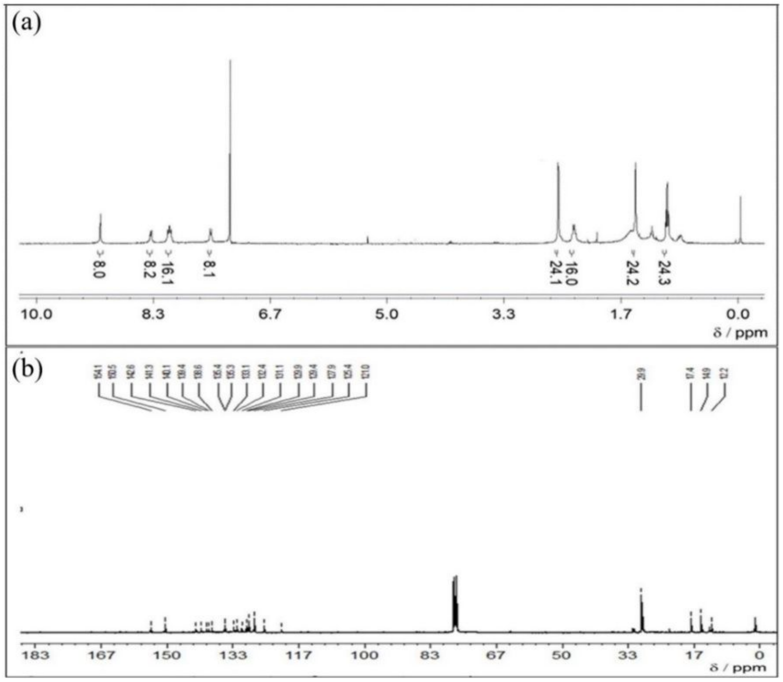

Figure S1. (a) ^{1}H NMR ($CDCl_3$, 400 MHz), (b) ^{13}C NMR ($CDCl_3$, 100 MHz) of F_6B_4P

Maldi-Tof Mass: m/z Calcd for [M+]: 2127.079; found: 2127.283

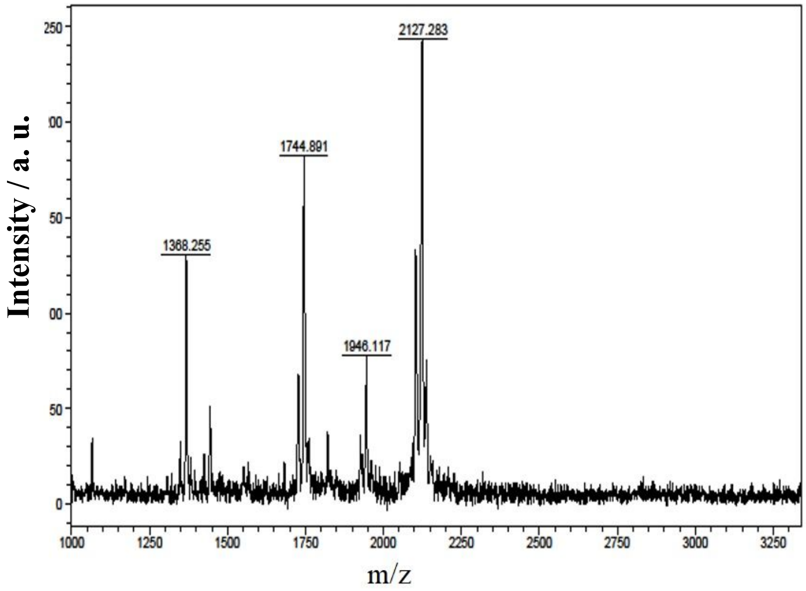

Figure S2. Maldi-TOF mass spectrum of F_6B_4P.

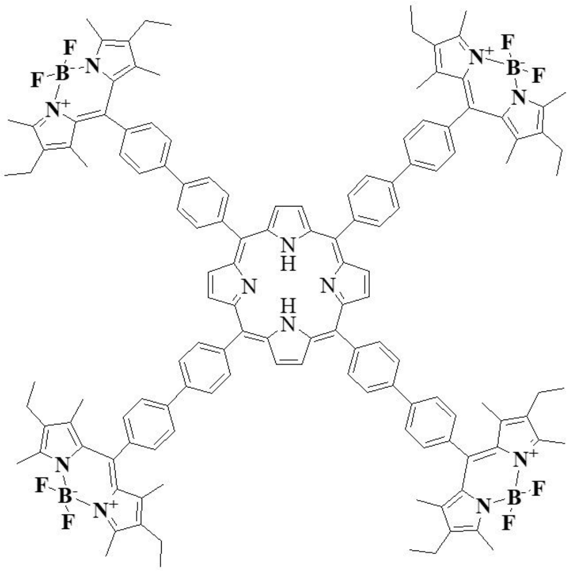

Figure S3. Chemical structure of the PRF.

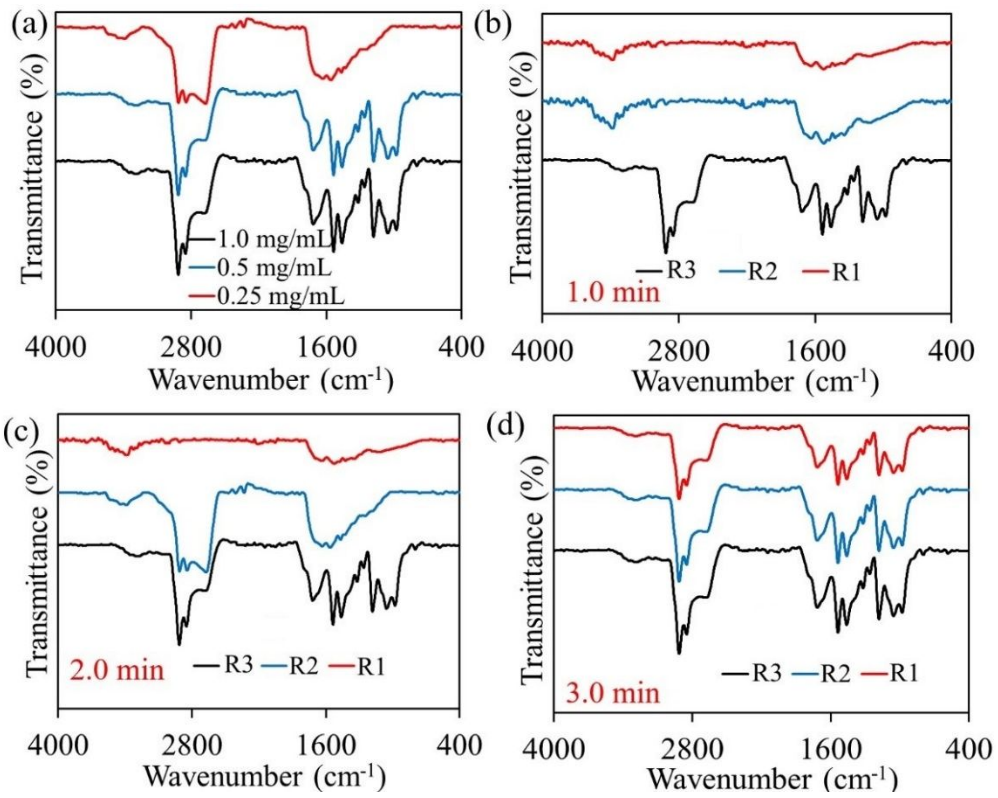

Figure S4. FTIR spectra of the R3 of GGSM impregnated in PRF solutions prepared at different concentrations for 1 min. FTIR spectra of R1, R2, and R3 of PRF/GGSMs prepared by modification of GGSM with PRF for (b) 1 min, (c) 2 min, (d) 3 min.

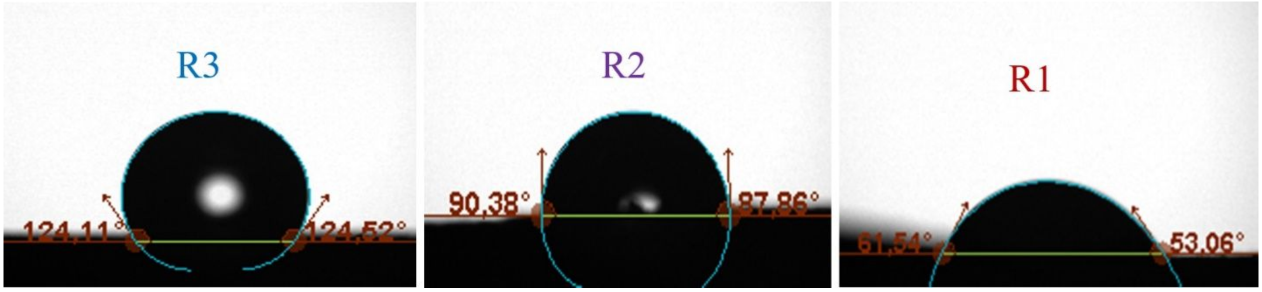

Figure S5. Water contact angle measurements for three different regions of GGSM prepared under optimum conditions.

Table S1. Atomic % ratios of R1, R2, and R3 regions of PRF/GGSM.

Regions of PRF/GGSM	C (%)	O (%)	N (%)	B (%)	F (%)
R1	62	38	-	-	-
R2	66	22	4	5	3
R3	68	14	6	7	5

The weight (ηw, %) and volume (ηV, %) swelling ratios of PRF/GGSM:

Weight swelling ratio (ηw, %) of PRF/GGSM was measured by immersing pre-weighed dry samples in deionized water at various temperatures (20, 30, 40, and 50 °C) until equilibrium. After all the material was completely saturated with water, excess surface water was removed with filter paper and the fully swollen sample was weighed. η_w was calculated from the following equation [1],

$$\eta_W = \frac{m_s - m_d}{m_d} x100 \qquad \text{(Equation S1)}$$

where m_s is the weight of the swollen state of the sample at equilibrium and m_d is the weight of the dry state of the sample. Table S2 presents the η_w of PRF/GGSM at different temperatures. Then, the volume swelling ratio (η_V, %) of the PRF/GGSM was determined by measuring the sample size before and after swelling with water. η_V was calculated from the following equation;

$$\eta_V = \frac{V_s - V_d}{V_d} x100 \qquad \text{(Equation S2)}$$

where V_s is the volume of the swollen state of the sample at equilibrium and V_d is the volume of the dry state of the sample.

Table S2. Weight swelling ratios (η_W, %) and volumetric swelling ratios (η_V, %) of PRF/GGSM at 20-50 °C.

	**PRF/GGSM**	
**Temperature (°C)**	η_W,%	η_V,%
20	1580	485
30	1536	469
40	1496	445
50	1458	418

Thermal conductivity of PRF/GGSM:

Thermal conductivity of PRF/GGSM were measured by cutting them in dimensions of 2.0 cm x 2.0 cm x 2.0 cm and sandwiching it between two glass layers dry and wet. The prepared "Sandwich" was placed between a heat source (ceramic plate warmer) and a cold source (ice water bath). The temperature distribution along the cross-section of the sandwich structure was monitored using an IR camera. Thermal conductivity was determined using the Fourier equation.

$$q' = K\frac{dT}{dx} \qquad \text{(Equation S3)}$$

where q' is the thermal conductivity per unit area, dT is the temperature difference, dx is the width of the sample and K is the thermal conductivity of the glass. The conductivity of glass is 1.05 $Wm^{-1}K^{-1}$ (for 3 mm glass thickness). The thermal conductivity calculation is based on the assumptions that the sample and glass slides are subjected to the same heat flux and that the emissivity coefficients of the sample and the glass sheet are both 0.9.[2,3]

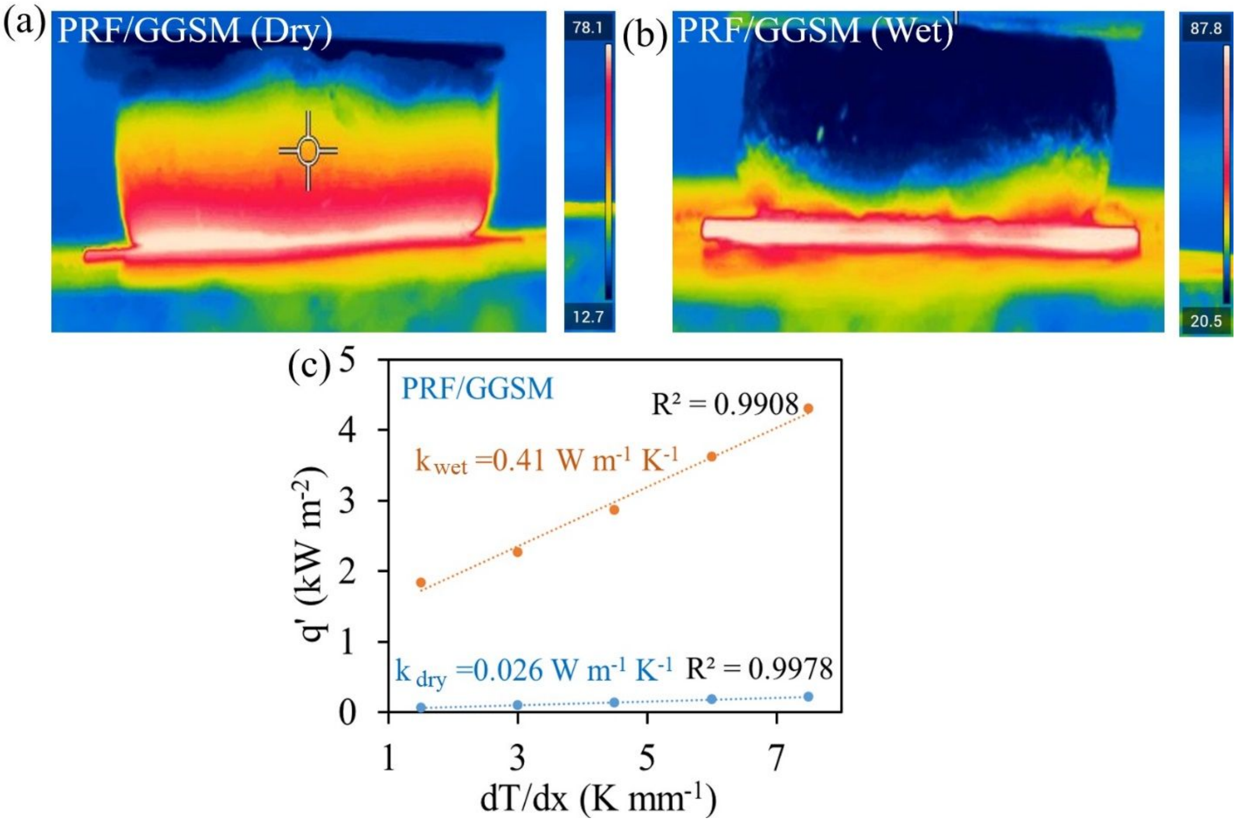

Figure S6. Thermal camera images of (a) wet and (b) dry PRF/GGSM. (c) Thermal conductivity of PRF/GGSM.

Table S3. Thermal conductivity values of PRF/GGSM in wet and dry conditions.

**Material**	$\mathbf{k_{dry}}$ (W m^{-1} K^{-1})	$\mathbf{k_{wet}}$ (W m^{-1} K^{-1})
PRF/GGSM	0.027	0.42

Solar thermal conversion efficiency of PRF/GGSM:

The efficiency of solar thermal conversion is expressed as follows:

$$\eta = \frac{m.h_{LV}}{l} \qquad \text{(Equation S4)}$$

where η is the solar vapor conversion efficiency, m is the evaporation rate, h_{LV} is the sum of the total enthalpy of sensible heat and the phase change enthalpy of the liquid, and I is the energy of sunlight.[4] When water molecules pass through in a porous medium, they are more likely to

evaporate in clusters rather than as single molecules [5]. Therefore, the evaporation enthalpy of water on the PRF/GGSM surface is lower than that of water.[6]

The enthalpies of water evaporation on the surfaces of sponge materials were calculated as follows:

$$m_{su}.h_{su} = m_{sm}.h_{sm} \qquad \text{(Equation S5)}$$

Here, m_{su} and m_{sm} are the evaporation rates of the water without any material on it and the water on the sponge material (SM) surface, respectively, and h_{su} and h_{sm} are the enthalpy of water and the water on the SM surface, respectively.[4] Using Equation 5, the evaporation enthalpy for PRF/GGSM was calculated as and 1440±22 J g^{-1} (The evaporation enthalpy of water assumed as 2450 J g^{-1} at 25 °C). When GGSM was modified with PRF, the enthalpy of evaporation decreased [7], attributed to the fact that supramolecules can transfer heat energy more quickly and thus bulk water evaporation.

Heat losses for the designed SSG system:

The following was calculated for the heat losses in Figure 7a.

i. Radiation.

Radiation loss was calculated using the Stefan−Boltzmann equation and 1 kW m^{-2} solar energy.[8]

$$ER = \varepsilon A\sigma(T^4 - T\infty^4) \qquad \text{(Equation S6)}$$

where ER is the heat flux, ε is the emission (assumed as 0.97), A is the surface area (13.5 cm^2), σ is the Stefan−Boltzmann constant (5.67×10^{-8} W m^{-2} K^{-4}), T is the maximum temperature (max. 77 °C) and T∞ is the ambient temperature during the experiment (average 20 °C). Thus, according to the Stefan−Boltzmann equation, the radiation losses for two different prepared systems were calculated and presented in Table S4.

ii. Convection.

Convection loss was calculated by Newton's law of cooling.[8]

$$Q = Ah(T - T\infty) \qquad \text{(Equation S7)}$$

where Q is the heat, h is the convection heat transfer coefficient, and A is the surface area (13.5 cm^2). The convection heat transfer coefficient is about 5 W m^{-2} K^{-1}. Using Equation S7, radiation losses were calculated for two systems and presented in Table S4.

iii. Transmission.

Transmission loss was calculated with the basic transmission law.[8]

$$Q = Cm\Delta T \qquad \text{(Equation S8)}$$

where Q is the heat, C is the specific heat capacity of water (4.2 J g^{-1} °C^{-1}), m is the weight of water (□30 g), and ΔT is how much the water temperature rises in 3600 seconds. Radiation losses for

two systems were calculated by Equation S8 and presented in Table S4.

Table S4. Heat transfer % of PRF/GGSM used in the SSG system.

	**Evaporation (%)**	**Radiation (%)**	**Convection (%)**	**Transmission (%)**
PRF/GGSM	96	1.9	1.2	0.9

Table S5. Water evaporation rate and solar thermal conversion efficiency of PRF/GGSM for water with different contents under 1 sunlight and after 1 h distillation.

	PRF/GGSM	
	Evaporation Rate (kg/m^2h)	% Efficiency
Pure water	3.81	96.1
Acidic water	3.77	95.7
Alkaline water	3.78	94.8
Saltwater	3.81	95.7
Heavy metal + water	3.79	95.3
Dye + water	3.78	94.9
**Average values**	**3.79**	**95.3**
**Standard deviation%**	**3.43**	**0.42**

Table S6. Ion amount after distillation of the solution containing 3.5% NaCl solution and 0.1% Na^+, K^+, Mg^{+2}, and Ca^{+2} ions with PRF/GGSM.

Material	3.5% NaCl Solution Na^+ ion (ppm)	Solution containing 0.1% Na^+, K^+, Mg^{+2}, and Ca^{+2}			
		Na^+ (ppm)	K^+ (ppm)	Mg^{+2} (ppm)	Ca^{+2} (ppm)
PRF/GGSM	100	0.9	1.1	2.2	3.0
Initially, 1275 ppm Na^+ ion and 900 ppm Na^+, 935 ppm K^+, 550 ppm Mg^{+2}, and 905 ppm Ca^{+2} were detected in the 3.5% NaCl and 0.1% ion solutions.					

Table S7. Times obtained for 10 mL of distillate from solutions containing salty, heavy metal, and dye with PRF/GGSM and GGSM.

Material	Salt removal	Heavy metal removal	Dye removal
	Time to obtain 10 mL of distillate (h)		
PRF/GGSM	4 .0	4.2	3.9
GGSM	8.2	8.4	7.9
Experiments were carried out with the 6.75 cm^{-2} surface area of the materials under 10 sunlight (1000 kW m^{-2}).			

Table S8. The change in the amounts of some ions in desalination with PRF/GGSM from seawater.

Samples	PRF/GGSM		
	Na (ppm)	Mg (ppm)	Ca (ppm)
Mediterranean seawater	12571	1656	132
Distilled Mediterranean seawater	5.2	1.2	1.5
Black Sea seawater	5571	751	67
Distilled Black Sea seawater	8.3	0.2	0.8

Table S9. Heavy metal ion amounts detected after distillation of the solution containing 225 mg/mL Pb^{+2}, 100 mg/mL, Cu^{+2}, 90 mg/mL, Ni^{+2}, 75 ppm Cr^{+3}, 150 mg/mL Zn^{+2} ions with PRF/GGSM.

Material	Heavy metal ions				
	Pb^{+2} (ppm)	Cu^{+} (ppm)	Ni^{+2} (ppm)	Cr^{+2} (ppm)	Zn^{+2} (ppm)
PRF/GGSM	0.15	0.23	0.12	0.11	0.14

Initially, a solution containing 225 mg/mL Pb^{+2}, 100 mg/mL Cu^{+2}, 90 mg/mL Ni^{+2}, 75 ppm Cr^{+3}, 150 mg/mL Zn^{+2} ion was used.

(a)

(b)

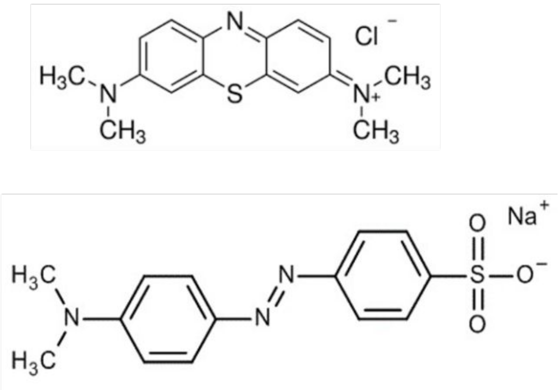

Figure S7. Chemical structure of (a) MB and (b) MO.

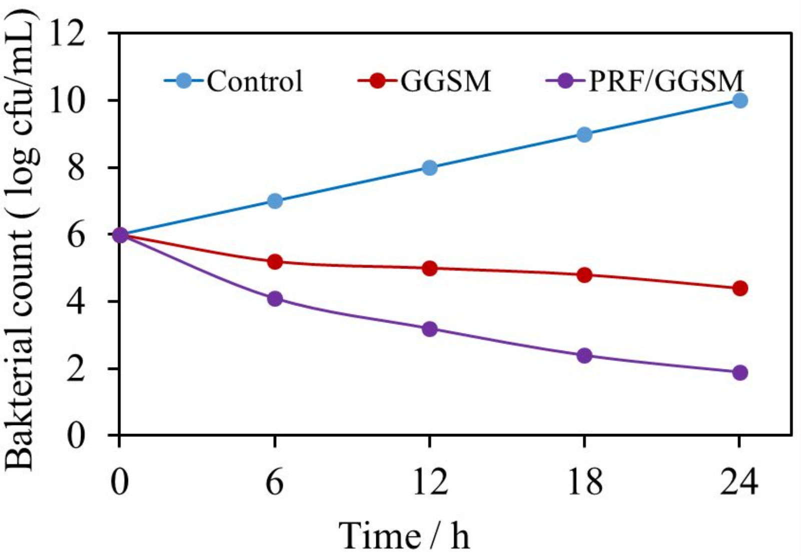

Figure S8. Antimicrobial activities of PRF/GGSM and GGSM.

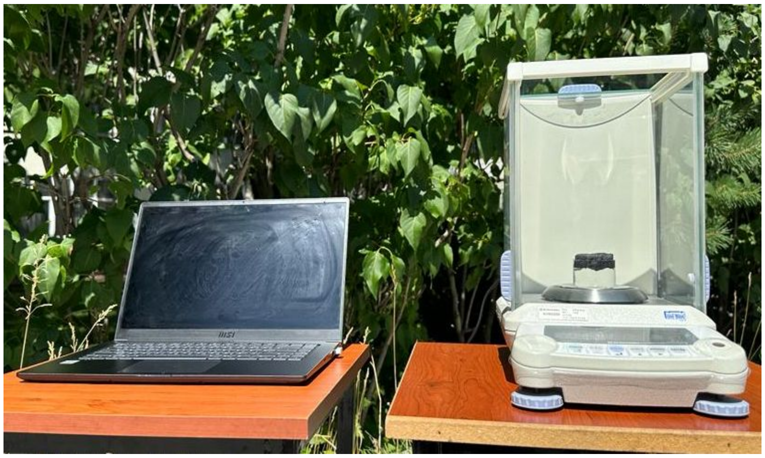

Figure S9. Photograph of solar steam generator application under daylight.

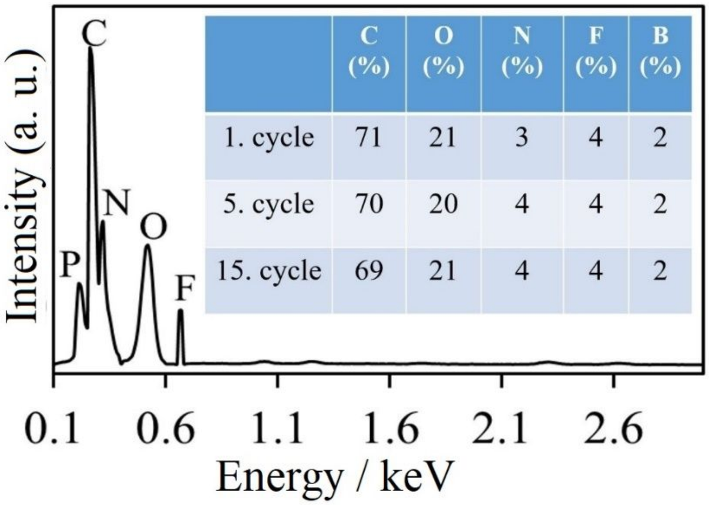

Figure S10. EDX spectrum after the 15th consecutive cycle of PRF/GGSM for desalination without considering the adsorbed salt ions. Inset: Table of the atomic ratios after the 1st, 5th, and 15th cycles of PRF/GGSM.

Table S10. The comparison of PRF/GGSM with various materials on SSG performance under 1 sun.

Material	Evaporation rate (kg/m^{2}h)	Efficiency %	Water adsorbing part	Photothermal material	Ref.
pPEGDA−PAN	1.40	91	Cellulose foam	Polyethyleneglycol -Polyaniline	1
Fe_2O_3/CNT/Ni nanocomposite	1.48	82	Ni foam	Fe_2O_3/CNT	9
PAN/GO	2.27	92	GO	PAN	10
PTCNFA	2.89	90	Nanofibrous gel	Nanofibrous gel	11
(CNF/PLA/PANI)	1.58	90	CNF/PLA	polylactic acid/polyaniline	12
3D AFA	1.43	86	3D fiber gel	3D fiber gel	13
MBCF	1.49	96	Carbon foam	Molybdenum sulfide	14
CNF/PLA/PANI	1.58	90	Cellulose nonofibril	Polylactic acid-Polyaniline	15
MS-PP	2	91	Melamine foam	polypyrrole	16
Janus VA-MXA	1.46	87	MXene gel	Fluoronized alkyl silane layer	17
GGSM	1.79	57	Graphene	Graphene	7
PRF/GGSM	3.8	92	Graphene	Graphene-PRF	This work

References

(1) Yin, X.; Zhang, Y.; Guo, Q.; Cai, X.; Xiao, J.; Ding, Z.; Yang, J. Macroporous Double-Network Hydrogel for High-Efficiency Solar Steam Generation under 1 Sun Illumination. *ACS Appl. Mater. Interfaces* **2018**, *10* (13), 10998–11007. https://doi.org/10.1021/acsami.8b01629.

(2) Liu, K. K.; Jiang, Q.; Tadepalli, S.; Raliya, R.; Biswas, P.; Naik, R. R.; Singamaneni, S. Wood-Graphene Oxide Composite for Highly Efficient Solar Steam Generation and Desalination. *ACS Appl. Mater. Interfaces* **2017**, *9* (8), 7675–7681.

https://doi.org/10.1021/acsami.7b01307.

(3) Xu, Y.; Wang, J.; Yu, F.; Guo, Z.; Cheng, H.; Yin, J.; Yan, L.; Wang, X. Flexible and Efficient Solar Thermal Generators Based on Polypyrrole Coated Natural Latex Foam for Multimedia Purification. *ACS Sustain. Chem. Eng.* **2020**, *8* (32), 12053–12062. https://doi.org/10.1021/acssuschemeng.0c03164.

(4) Kim, C.; Shin, D.; Baitha, M. N.; Ryu, Y.; Urbas, A. M.; Park, W.; Kim, K. High-Efficiency Solar Vapor Generation Boosted by a Solar-Induced Updraft with Biomimetic 3D Structures. *ACS Appl. Mater. Interfaces* **2021**, *13* (25), 29602–29611. https://doi.org/10.1021/acsami.1c05883.

(5) Gong, F. (Frank); Li, H.; Wang, W.; Huang, J.; Xia, D. (David); Liao, J.; Wu, M.; Papavassiliou, D. V. Scalable, Eco-Friendly and Ultrafast Solar Steam Generators Based on One-Step Melamine-Derived Carbon Sponges toward Water Purification. *Nano Energy* **2019**, *58* (November 2018), 322–330. https://doi.org/10.1016/j.nanoen.2019.01.044.

(6) Zhao, F.; Zhou, X.; Shi, Y.; Qian, X.; Alexander, M.; Zhao, X.; Mendez, S.; Yang, R.; Qu, L.; Yu, G. Highly Efficient Solar Vapour Generation via Hierarchically Nanostructured Gels. *Nat. Nanotechnol.* **2018**, *13* (6), 489–495. https://doi.org/10.1038/s41565-018-0097-z.

(7) Erçarıkcı, E.; Topçu, E.; Kudaş, Z.; Aksu, Z.; Alanyalıoğlu, M.; Dağcı Kıranşan, K. An Effective Material for Solar Steam Generation Applications: Gradient Graphene Sponge. *Mater. Today Sustain.* **2024**, *26* (December 2023), 1–11. https://doi.org/10.1016/j.mtsust.2024.100701.

(8) Yang, Y.; Zhao, R.; Zhang, T.; Zhao, K.; Xiao, P.; Ma, Y.; Ajayan, P. M.; Shi, G.; Chen, Y. Graphene-Based Standalone Solar Energy Converter for Water Desalination and Purification. *ACS Nano* **2018**, *12* (1), 829–835. https://doi.org/10.1021/acsnano.7b08196.

(9) Han, S.; Yang, J.; Li, X.; Li, W.; Zhang, X.; Koratkar, N.; Yu, Z. Z. Flame Synthesis of Superhydrophilic Carbon Nanotubes/Ni Foam Decorated with Fe2O3 Nanoparticles for Water Purification via Solar Steam Generation. *ACS Appl. Mater. Interfaces* **2020**, *12* (11), 13229–13238. https://doi.org/10.1021/acsami.0c00606.

(10) Wang, L.; Liu, C.; Wang, H.; Xu, Y.; Ma, S.; Zhuang, Y.; Xu, W.; Cui, W.; Yang, H. Three-Dimensional Wood-Inspired Bilayer Membrane Device Containing Microchannels for Highly Efficient Solar Steam Generation. *ACS Appl. Mater. Interfaces* **2020**, *12* (21), 24328–24338. https://doi.org/10.1021/acsami.0c04740.

(11) Mei, T.; Chen, J.; Zhao, Q.; Wang, D. Nanofibrous Aerogels with Vertically Aligned Microchannels for Efficient Solar Steam Generation. *ACS Appl. Mater. Interfaces* **2020**, *12* (38), 42686–42695. https://doi.org/10.1021/acsami.0c09518.

(12) Xu, W.; Xing, Y.; Liu, J.; Wu, H.; Cui, Y.; Li, D.; Guo, D.; Li, C.; Liu, A.; Bai, H. Efficient Water Transport and Solar Steam Generation via Radially, Hierarchically Structured Aerogels. *ACS Nano* **2019**, *13* (7), 7930–7938. https://doi.org/10.1021/acsnano.9b02331.

(13) Li, H.; Li, H.; Li, H.; Wen, H.; Li, J.; Li, J.; Huang, J.; Huang, J.; Huang, J.; Wang, D.;

Tang, B. Z. Doping AIE Photothermal Molecule into All-Fiber Aerogel with Self-Pumping Water Function for Efficiency Solar Steam Generation. *ACS Appl. Mater. Interfaces* **2020**, *12* (23), 26033–26040. https://doi.org/10.1021/acsami.0c06181.

(14) Zhang, X.; Wu, G.; Yang, X. C. MoS2 Nanosheet−carbon Foam Composites for Solar Steam Generation. *ACS Appl. Nano Mater.* **2020**, *3* (10), 9706–9714. https://doi.org/10.1021/acsanm.0c01712.

(15) Li, S.; He, Y.; Guan, Y.; Liu, X.; Liu, H.; Xie, M.; Zhou, L.; Wei, C.; Yu, C.; Chen, Y. Cellulose Nanofibril-Stabilized Pickering Emulsion and in Situ Polymerization Lead to Hybrid Aerogel for High-Efficiency Solar Steam Generation. *ACS Appl. Polym. Mater.* **2020**, *2* (11), 4581–4591. https://doi.org/10.1021/acsapm.0c00674.

(16) Chen, T.; Wu, Z.; Liu, Z.; Aladejana, J. T.; Wang, X. (Alice); Niu, M.; Wei, Q.; Xie, Y. Hierarchical Porous Aluminophosphate-Treated Wood for High-Efficiency Solar Steam Generation. *ACS Appl. Mater. Interfaces* **2020**, *12* (17), 19511–19518. https://doi.org/10.1021/acsami.0c01815.

(17) Zhang, Q.; Yi, G.; Fu, Z.; Yu, H.; Chen, S.; Quan, X. Vertically Aligned Janus MXene-Based Aerogels for Solar Desalination with High Efficiency and Salt Resistance. *ACS Nano* **2019**, *13* (11), 13196–13207. https://doi.org/10.1021/acsnano.9b06180.
